# Mitochondrial DNA Sequence and Lack of Response to Anoxia in the Annual Killifish *Austrofundulus limnaeus*

**DOI:** 10.3389/fphys.2016.00379

**Published:** 2016-08-31

**Authors:** Josiah T. Wagner, Florisela Herrejon Chavez, Jason E. Podrabsky

**Affiliations:** Department of Biology, Center for Life in Extreme Environments, Portland State UniversityPortland, OR, USA

**Keywords:** annual killifish, anoxia, mitochondria, citrate synthase, embryo, mitochondrial genome

## Abstract

The annual killifish *Austrofundulus limnaeus* inhabits ephemeral ponds in regions of Venezuela, South America. Permanent populations of *A. limnaeus* are maintained by production of stress-tolerant embryos that are able to persist in the desiccated sediment. Previous work has demonstrated that *A. limnaeus* have a remarkable ability to tolerate extended periods of anoxia and desiccating conditions. After considering temperature, *A. limnaeus* embryos have the highest known tolerance to anoxia when compared to any other vertebrate yet studied. Oxygen is completely essential for the process of oxidative phosphorylation by mitochondria, the intracellular organelle responsible for the majority of adenosine triphosphate production. Thus, understanding the unique properties of *A. limnaeus* mitochondria is of great interest. In this work, we describe the first complete mitochondrial genome (mtgenome) sequence of a single adult *A. limnaeus* individual and compare both coding and non-coding regions to several other closely related fish mtgenomes. Mitochondrial features were predicted using MitoAnnotator and polyadenylation sites were predicted using RNAseq mapping. To estimate the responsiveness of *A. limnaeus* mitochondria to anoxia treatment, we measure relative mitochondrial DNA copy number and total citrate synthase activity in both relatively anoxia-tolerant and anoxia-sensitive embryonic stages. Our cross-species comparative approach identifies unique features of *ND1, ND5, ND6*, and *ATPase-6* that may facilitate the unique phenotype of *A. limnaeus* embryos. Additionally, we do not find evidence for mitochondrial degradation or biogenesis during anoxia/reoxygenation treatment in *A. limnaeus* embryos, suggesting that anoxia-tolerant mitochondria do not respond to anoxia in a manner similar to anoxia-sensitive mitochondria.

## Introduction

Mitochondria are intracellular organelles that play a central and essential role in basic eukaryotic cell biology by regulating metabolism, the cell cycle, and cell survival or death. The organelle is best known for synthesizing the majority of cellular adenosine triphosphate (ATP) by oxidative phosphorylation (OXPHOS). Synthesis of ATP through mitochondrial OXPHOS is completely dependent on a constant availability of oxygen as the terminal electron acceptor. In most vertebrates, prolonged exposure to anoxia typically causes mitochondrial dysfunction and eventually cell death by necrosis or apoptosis (Johnston et al., 2001; Verma et al., 2002; Levraut et al., 2003). However, some aquatic vertebrates are able to tolerate extensive periods of anoxia. Of these anoxia-tolerant vertebrates, embryos of the annual killifish *Austrofundulus limnaeus* (Meyers) have a tolerance of anoxia that is approximately two orders of magnitude greater than any other vertebrate species yet examined (Podrabsky et al., 2007, 2012b). Development in *A. limnaeus* has a predictable and unique phenotype that has been well-characterized. Thus, it is an ideal model organism for studying endogenous mechanisms that may facilitate extreme stress tolerance.

*A. limnaeus* (Family Rivulidae, Order Cyprinodontiformes) is commonly found in ephemeral habitats of the Maracaibo basin of Venezuela (Podrabsky et al., 1998; Hrbek et al., 2005). Due to seasonal fluctuations in water and oxygen availability, embryos of *A. limnaeus* embryos likely experience hypoxic or anoxic conditions as a regular part of their embryonic development (Podrabsky et al., 1998, 2015). In preparation for the seasonal desiccation of their habitat, *A. limnaeus* embryos enter into a state of developmental arrest and deep metabolic depression known as diapause. Diapause may occur at 3 distinct stages of development in annual killifish embryos, termed diapause I, II, and III (Wourms, 1972a,b,c). Although rare or absent in our lab stock, diapause I (DI) can occur early in development after completion of epiboly but prior to the formation of the embryonic axis (Wourms, 1972c). Diapause II (DII) occurs in the long-somite embryo approximately midway through development, just prior to the major phases of organogenesis, and appears to be the most stress-resistant diapause stage (Wourms, 1972a,c; Podrabsky et al., 2001, 2007, 2015). Finally, embryos may arrest as a late pre-hatching embryo in diapause III (DIII) rather than immediately hatching (Wourms, 1972a).

Anoxia tolerance peaks in *A. limnaeus* approximately midway through development during DII; this tolerance is retained for the first 4–6 days of post-diapause II (PDII) development and is then subsequently lost by the time embryos enter DIII (Podrabsky et al., 2007, 2012b). The mechanisms by which *A. limnaeus* embryos are able to tolerate anoxia are not completely known. However, previous work has suggested that during diapause, a combination of reduced ATP consumption through depressed protein synthesis, extremely low levels of OXPHOS activity, and depressed heart rate likely all contribute to the anoxia tolerant phenotype (Podrabsky and Hand, 1999, 2000; Duerr and Podrabsky, 2010). If exposed to anoxia during active development, embryos enter into a reversible state of quiescence and within hours of exposure experience a dramatic drop in ATP levels (Podrabsky et al., 2007, 2012a). Prolonged anoxia and acute deceases in ATP are typically followed by mitochondrial-mediated cell death by apoptosis in other vertebrates, yet *A. limnaeus* embryos do not appear to follow the same trajectory (Hand and Menze, 2008; Podrabsky et al., 2012a; Meller and Podrabsky, 2013). These studies suggest that *A. limnaeus* embryos have an atypical response to anoxia that likely involves unique mitochondrial physiology.

The process of OXPHOS is inherently complicated, requiring coordination between nuclear and mitochondrial proteins. Changes in mitochondrial DNA (mtDNA) sequence and copy number can have profound effects on mitochondrial protein function and expression. These changes can often have effects on mitochondrial physiology and subsequently affect the ability of an organism to cope with oxygen stress (Bratic and Larsson, 2013; Kowald and Kirkwood, 2014). Therefore, understanding how the *A. limnaeus* mitochondrial genome (mtgenome) may differ from less stress-tolerant species, and the phylogenetic relationship to other species, may provide insight into vertebrate mitochondrial evolution under the unique and intense selection pressures imposed by life in an ephemeral aquatic habitat. Additionally, little is known about the regulation of *A. limnaeus* embryonic mitochondrial biomass during anoxia, specifically whether or not mitochondrial genomes are stabilized or degraded. Previous work has suggested that in anoxia- and hypoxia-sensitive mammalian models, exposure of cells to long-term hypoxic conditions can result in mitochondrial autophagy (mitophagy) while acute anoxia followed by reoxygenation appears to result in mitochondrial biogenesis (Yin et al., 2008; Zhang et al., 2008; Youle and Van Der Bliek, 2012). As a part of these previous studies, measurement of mitochondrial:genomic DNA ratios and/or total citrate synthase (CS) activity were used to give an indication of mitochondrial degradation or biogenesis during normal and low oxygen conditions. We apply a similar strategy to explore the responsiveness of *A. limnaeus* embryonic mitochondria to sublethal anoxia/reoxygenation by measuring relative mtDNA content using quantitative PCR (qPCR) and total CS activity in post-diapause II embryos.

This work describes the first complete mitochondrial genome sequence of *A. limnaeus* (Strain: Quisiro) as assembled from a single adult male. We also use RNAseq mapping to infer post-transcriptional polyadenylation of primary mitochondrial transcripts. We discuss the unique features of the *A. limnaeus* mtgenome annotation when compared to several other species from the Order Cyprinodontiformes. To determine potential shifts in mitochondrial physiology, we measure relative mtDNA content and total CS activity following anoxia/reoxygenation in both relatively anoxia-tolerant [Wourms' stage (WS) 36, 4 days post-diapause II (dpd), LD_50_ anoxia 65 = days] and anoxia-sensitive (WS 40, 12 dpd, LD_50_ anoxia = 6 days; WS 42, 22 dpd, LD_50_ anoxia = 12.1 h) embryos (Podrabsky et al., 2007). Potential implications for understanding the role of mitochondria in the anoxia tolerance of *A. limnaeus* are discussed.

## Materials and methods

### Husbandry of adults and maintenance of embryos

Adult *A. limnaeus* and their embryos were cared for as previously described by Podrabsky (1999) and in accordance with an approved Portland State University Institutional Animal Care and Use Committee protocol (PSU protocol #33). Briefly, mating pairs were allowed access to spawning trays containing 1–2 cm of 500 μm diameter soda-lime glass beads (30/40 mesh glass bead blasting media, Kramer Industries, Inc., Piscataway, NJ, USA) for 2 h. Embryos were separated from the glass beads by straining through a 1.5 mm mesh and were collected using a wide-mouthed plastic pipette. Unfertilized eggs were discarded. For the first 3 d of development, embryos were kept in embryo medium formulated to mimic the ionic composition of their native ponds (10 mmol l^−1^ NaCl, 2.14 mmol l^−1^ MgCl_2_, 0.8 mmol l^−1^ CaCl_2_, 0.14 mmol l^−1^ KCl, 0.0013 mmol l^−1^MgSO_4_) containing 0.0001% methylene blue to suppress fungal growth (Podrabsky et al., 1998; Podrabsky, 1999). At 4 days post-fertilization, embryos were treated with two 5 min washes of a 0.01% solution of sodium hypochlorite (Clorox Bleach) in embryo medium and transferred to embryo medium containing 10 mg l^−1^ gentamicin sulfate (Podrabsky, 1999). Embryo medium was changed daily and embryos were incubated at 25°C in darkness until sorting.

### Total genomic DNA extraction of adult tissues

#### DNA extraction of adult tissues for illumina sequencing

The tissue used for DNA sequencing was derived from a single male *A. limnaeus* at 3 months post-hatch. The laboratory stock this individual was sampled from has been maintained since 1995 and originated from near Quisiro, Maracaibo basin, Venezuela. The fish was euthanized by immersion in an ice bath for ~5 min followed by cervical transection. Liver, white muscle (with skin and scales removed), and brain tissue were removed and transferred to DNA extraction buffer [10 mmol l^−1^ Tris-HCl pH = 8.0, 100 mmol l^−1^ EDTA, 0.5% SDS, 200 μg ml^−1^ Proteinase K (Thermo Scientific, Waltham, MA, USA, #EO0491)] at a ratio of 1 mg tissue per 10 μl buffer. Tissues were gently homogenized using a Teflon pestle in 1.5 ml microcentrifuge tubes and incubated for 3 h at 50°C with agitation every 20–30 min. DNA was extracted by briefly vortexing the homogenates with 1 vol phenol (equilibrated with 10 mmol l^−1^ Tris-HCl, pH = 8.0, 1 mmol l^−1^ EDTA) followed by centrifugation at 5000 × g for 10 min at 4°C. The aqueous phases were collected using a wide-bore pipette and extracted with phenol as described above. Following the second phenol extraction, the aqueous phases containing the DNA were extracted a third time by gentle mixing with 1 volume of chloroform:phenol (1:1) followed by centrifugation at 5000 × g for 10 min at 4°C. The aqueous phase was collected and the DNA precipitated by the addition of NaCl (final concentration of 200 mmol l^−1^) and 2 volumes of 100% ethanol (EtOH) followed by mixing by gentle inversion. The DNA was pelleted by centrifugation at 16,000 × *g* for 10 min at 4°C. The DNA pellet was washed twice with 1 ml 70% EtOH. After each wash the DNA was pelleted by centrifugation at 16,000 × g for 5 min at 4°C. The air-dried DNA pellet was resuspended in 500 μl of RNAse buffer (10 mmol l^−1^ Tris-HCl, pH = 8, 5 mmol l^−1^ EDTA) with 100 μg ml^−1^ DNAse-free RNAse A (Thermo Scientific #EN0531) and incubated at 37°C for 45 min with occasional tube inversion. The samples were extracted twice more as described above with chloroform:phenol (1:1) followed by precipitation using 0.1 vol of 3 mol l^−1^ sodium acetate pH = 5.2, and 2 vol of 100% EtOH. The DNA was pelleted by centrifugation at 16,000 × g for 10 min at 4°C and the pellet was washed twice with 1 ml 70% EtOH as described above. The DNA pellets were dried briefly at room temperature and resuspended in 100 μl of buffer EB (10 mmol l^−1^ Tris-HCl pH = 8.5, Qiagen, Hilden, Germany, #19086) at 37°C with occasional agitation until pellets dissolved completely.

#### DNA quantification and quality assessment

Total genomic DNA concentrations and A_260_/A_280_ ratios were determined using the Infinite M200 Pro plate reader equipped with a NanoQuant plate (Tecan, San Jose, CA, USA) using 2 μL of sample and default software settings (i-control software, Tecan). We considered A_260_/A_280_ ratios between 1.9 and 2.1 to be of acceptable DNA purity. DNA integrity was determined by 1% agarose gel electrophoresis of 1 μg of total DNA and observation of high-molecular weight DNA above 20 kb.

### DNA sequencing and read quality control

#### Illumina sequencing

DNA sequencing libraries from the mixed tissue sample were prepared and sequenced at the University of Oregon High Throughput DNA Sequencing and Genomics facility. Purified DNA was sonicated to an average size of 170 bp and was prepared for sequencing using the Nextera library prep kit (Illumina, San Diego, CA, USA). Fragment libraries were sequenced on the Illumina Hi-Seq 2500 platform with 101 bp paired-end reads.

#### Sanger sequencing

Polymerase chain reaction (PCR) was used to amplify specific regions of the mitochondrial genome. PCR products were generated using total DNA from adult liver as the DNA template. PCR reactions (50 μl total volume) consisted of 50 ng total DNA, 10 pmol of forward and reverse primers (Table S1, Integrated DNA Technologies, Coralville, IA, USA), 0.25 U *Taq* polymerase (New England BioLabs, Ipswich, MA, USA #M0267L), in 1X ThermoPol buffer (New England BioLabs # M0267L). The reactions were heated for an initial 5 min at 95°C followed by 40 cycles of: 20 s at 95°C, 20 s at 60°C, and 5 min at 68°C. A single final elongation step of 10 min at 68°C followed the initial 40 cycles. PCR products were resolved via 1% agarose electrophoresis and product size was estimated by comparison to a DNA ladder (GeneRuler 1 kb Plus, Thermo Scientific #SM0312) using GelAnalyser (http://www.sequentix.de/). PCR products were then excised and cloned into the pGEM-T vector or sequenced directly (Table S2). Plasmids were heat-shock transformed into *E. coli* JM109 high-efficiency cells (Promega #L2001, Madison, WI, USA) as recommended by the manufacturer. Plasmids were purified using the Qiagen Miniprep kit and Sanger sequenced at Oregon Health and Science University using the primers indicated in Table S2.

#### Read quality control

Adapters were removed from Illumina reads using Trimmomatic version 0.36 (Bolger et al., 2014) and the included Illumina adapter list. Error-correction was performed using the Allpaths error correction module (Gnerre et al., 2011). Of the total read pool that passed adapter trimming and error-correction, 100 million forward and reverse reads were randomly chosen for mtgenome assembly (Table S3). Sanger reads were trimmed of vector contamination using the UniVec tool in the Geneious 6.1.8 suite (Biomatters, Auckland, New Zealand) and bases with a greater than 5% chance of error were trimmed (Kearse et al., 2012).

### Assembly and annotation of the *A. limnaeus* mitochondrial genome

The *A. limnaeus* mitochondrial genome was assembled using mitochondrial baiting and iterative mapping (MitoBIM) software on the default settings (Hahn et al., 2013). Input sequence consisted of 100 million post-trim forward reads (SRA #SRR2006331, Table S3). These reads were also a part of the read pool used to assemble the *A. limnaeus* nuclear genome (GenBank assembly accession: GCA_001266775.1). A short DNA sequence of *A. limnaeus* cytochrome *c* oxidase I (NCBI accession AF002589.1) was used to seed the initial baiting of mitochondrial reads. Geneious software (version 8.1.7) was used to generate and visualize subsequent alignments (Kearse et al., 2012). Sanger contigs from sequenced clones were assembled *de novo* and mapped to the mtgenome using Geneious (Table S2). Forward and reverse reads were mapped using the Geneious algorithm to determine coverage and assembly quality (max gap size 15 bp; minimum ≥90% overlap identity; multiple best matches randomly mapped; paired reads must match nearby; word length = 10). Single nucleotide polymorphisms (SNPs) were called using the Geneious Variant Caller with a minimum frequency of 10% and more stringent mapping settings (≥99.9% read mapping confidence; max gap size 15 bp; ≥90% identity; multiple best matches matched to none; paired reads must match nearby; word length = 10).

The resulting *A. limnaeus* mitochondrial genome was annotated using the MitoAnnotator pipeline (Iwasaki et al., 2013). Conserved sequence blocks (CSBs) were identified from consensus sequences derived from previously identified sequences (Broughton et al., 2001; Lee et al., 2001). The MITOS webserver was used to fold tRNAs into predicted cloverleaf structures (Bernt et al., 2013). To identify the *A. limnaeus* tRNA-Ser, the tRNA-Ser from *N. furzeri* was mapped to the *A. limneus* reference mtgenome using Geneious (50% identity minimum setting). Repetitive motif identification and clustering were obtained using RepFind (Betley et al., 2002).

### Mitochondrial genome investigations

#### Phylogenetic analysis

Complete mitochondrial genomes from several other Cyprinodontiform fishes were retrieved from the MitoFish database for comparative analysis (Table 1; Iwasaki et al., 2013). Mitochondrial genes in comparator species were identified using the MitoAnnotator pipeline as done with *A. limnaeus*. Individual coding genes without stops, rRNAs, and tRNAs were aligned using the MUSCLE alignment tool as part of the Geneious suite ver. 8.1.7 (Edgar, 2004; Kearse et al., 2012). Third codon positions were removed from alignments to avoid saturation bias (Kumazawa and Nishida, 1993). Poorly aligned regions were trimmed using the GBLOCKS webserver (http://molevol.cmima.csic.es/castresana/Gblocks_server.html) with default settings (Castresana, 2000). JModelTest 2 was used to estimate the best model of nucleotide substitution on the concatenated alignment (Darriba et al., 2012). The final alignment contained 10,875 nucleotide positions and phylogenetic relationships were inferred using RAxML-HPC 7.7.2 using settings for GTR + I + G, 1000 bootstrap replications, and partitioning by first and second codon positions, rRNAs, and tRNAs (Stamatakis, 2006). RAxML was run using the rapid-hill climbing algorithm that also returned the best-resulting maximum likelihood (ML) tree in a single step. *Oryzias latipes* (Order Beloniformes) was specified as an outgroup.

**Table 1:** **List of fish mitochondrial genomes used in this work**.

**Species**	**Family**	**Annual**	**Genbank accession**	**Total length (bp)**	**D-loop length (bp)^a^**	**Total GC%^b^**	**Coding GC%^c^**	**References**
*Austrofundulus limnaeus*	Rivulidae	Yes	KX371089	21,039	2284 (4647)	37.3	39.1	This work
*Nothobranchius furzeri*	Nothobranchiidae	Yes	NC_011814.1	19,527	2091 (3273)	38.6	38.4	Hartmann et al., 2011
*Austrolebias charrua*	Rivulidae	Yes	NC_028510.1	17,271	1349	36.8	37.4	Gutiérrez et al., 2015
*Kryptolebias marmoratus*	Rivulidae	No	NC_003290	17,329	887 (1682)	43.3	44	Lee et al., 2001
*Aplocheilus panchax*	Aplocheilidae	No	NC_011176	16,519	860	42.7	42.8	Setiamarga et al., 2008
*Cyprinodon rubrofluviatilis*	Cyprinodontidae	No	NC_009125	16,501	831	46.5	47.3	Crowl et al., unpublished
*Fundulus heteroclitus*	Fundulidae	No	NC_012312	16,526	865	39.7	38.9	Whitehead, 2009
*Fundulus diaphanus*	Fundulidae	No	NC_012361	16,531	867	38.9	38	Whitehead, 2009
*Fundulus grandis*	Fundulidae	No	NC_012377	16,524	866	40.4	39.9	Whitehead, 2009
*Fundulus olivaceus*	Fundulidae	No	NC_011380	16,509	853	42.2	42.3	Whitehead, 2009
*Gambusia affinis*	Poeciliidae	No	NC_004388	16,614	858	44.9	45.5	Miya et al., 2003
*Jordanella floridae*	Cyprinodontidae	No	NC_011387	16,177	-	45	45.2	Setiamarga et al., 2008
*Xenotoca eiseni*	Goodeidae	No	NC_011381	16,735	862	41.5	41.6	Setiamarga et al., 2008
*Xiphophorus hellerii*	Poeciliidae	No	NC_013089	16,635	857	47.8	49	Setiamarga et al., 2008
*Xiphophorus maculatus*	Poeciliidae	No	NC_011379	16,646	859	46.8	47.8	Setiamarga et al., 2008
*Oryzias latipes*	Adrianichthyidae	No	NC_004387	16,714	1073	44.5	45	Miya et al., 2003

*All species are from the Order Cyprinodontiformes except for O. latipes, which is Order Beloniformes*.

a *Total length of the D-loop region closest to the beginning of the annotated mitochondrion. If a D-loop is duplicated, the total of both D-loop regions is indicated in parenthesis. The mitochondrial sequence of J. floridae is incomplete and is missing part of the D-loop region*.

b *Total GC% of the entire mtgenome for each species*.

c *Total GC% of coding genes, not including stop codons*.

#### Codon usage and nucleotide composition

Codon usage tables and nucleotide position composition ratios of coding genes were obtained using CAIcal (Puigbò et al., 2008).

#### RNAseq mapping and polyadenylatyion calling

Illumina RNAseq reads derived from a pooled sample of 24 somite embryos reared at 30°C (SRA #SRR2032241) were used to identify mRNA expression of the *A. limnaeus* mtgenome in addition to polyadenylation signals. Sequenced reads were filtered on quality scores and trimmed for the presence of adapter sequences using Trimmomatic (Bolger et al., 2014) with the following settings: ILLUMINACLIP:2:30:7:1:true, SLIDINGWINDOW:5:15, LEADING:20, TRAILING:20, and MINLEN: 25. Following trimming, RNAseq reads had an average length of 97 bp, standard deviation of 11 bp, and a read length range of 25–100 bp. Filtered and trimmed reads were initially mapped to the annotated *A. limnaeus* mtgenome using Geneious software (version 8.1.7; max gap size = 5, max gaps per read 10%, ≥80% identity, map multiple best matches randomly) to estimate overall coverage of the mtgenome by RNAseq. To predict polyadenylation sites, slightly more stringent mapping settings were used that omitted reads with multiple best matches (≥99.9% read mapping confidence, no gaps, ≥80% identity, word length = 10). Similar to previous studies, we set a threshold requiring three or more non-template adenine nucleotides at a variant frequency of >4% to call a polyadenylated region at a putative 3′ transcript end (Marková et al., 2015).

#### RNA and DNA secondary structure predictions

The *A. limnaeus* mitochondrial origin of light-strand replication (Ori-L) and tRNA-Ser putative secondary structures were predicted using Geneious software ver 8.1.7 with default DNA energy mode settings at 25°C (Mathews et al., 2004 model).

#### Membrane protein topology predictions

The TOPCONS webserver was used for consensus predictions of *A. limnaeus* mitochondrial peptide membrane topology with default settings (Tsirigos et al., 2015). Secondary structure predictions were performed using PSIPRED v3.3 with default settings on the PSIPRED Protein Sequence Analysis Workbench webserver (Jones, 1999; Buchan et al., 2013). Bovine (*Bos taurus*) mitochondrial peptide sequences for ND1 (P03887), ND5 (Q85BD6), and ND6 (P03924) were downloaded from the UniProt database and used for comparison to *A. limnaeus* homologs (UniProt, 2015).

### qPCR analysis of normoxic and anoxia-treated *A. limnaeus* embryos

#### Anoxia treatment

Embryos were exposed to anoxia as previously described (Podrabsky et al., 2012a). To obtain PDII embryos, *A. limnaeus* embryos were staged at DII and diapause was experimentally terminated by placing dormant embryos in a 33°C incubator illuminated with 24 h illumination for 2 days before being returned to 25°C in the dark. PDII embryos were staged to one of three developmental stages using a dissecting scope: WS 36 (early organogenesis, 4 dpd), WS 40 (full overgrowth, 12 dpd), or WS 42 (early prehatching, 22 dpd; Wourms, 1972a). Prior to anoxia treatment, embryos were collected in petri dishes (100 × 15 mm plastic dishes) and excess embryo medium was removed using a pipette. Embryos were transferred into a Bactron III anaerobic chamber (Sheldon Manufacturing, Cornelius, OR) and rinsed three times with embryo medium that was previously purged for 30 min with N_2_ gas and allowed to equilibrate inside the anoxic chamber overnight. Anoxic conditions were maintained through the use of an anaerobic gas mixture (5% H_2_, 5% CO_2_, balance N_2_) and a palladium catalyst for the duration of anoxia exposure. Embryos at WS 36, 40, and 42 were sampled in normoxia (*t* = 0), after 24 h of anoxia, and after 24 h of aerobic recovery from anoxia (9 embryos per replicate). Unexposed normoxic embryos at 24 and 48 h were also sampled for parallel comparison. Diapause II embryos were sampled only in normoxia. Embryos were incubated at 25°C for the duration of the experiment in all treatments. Prior to sampling, embryos were collected onto a nylon mesh screen (100 μm mesh) and blotted dry using Kimwipes before being transferred into 1.5 ml microcentrifuge tubes and flash-frozen in liquid N_2_. Frozen embryos were stored at −80°C until DNA extraction.

#### Total genomic DNA extraction of embryos

Embryonic DNA was extracted using the DNeasy Blood and Tissue Kit (Qiagen, #69581). Embryos were homogenized on ice in microcentrifuge tubes using a Teflon pestle with 400 μl of kit provided lysis buffer ATL and 2 mg ml^−1^ of Proteinase K (Thermo Scientific, #EO0491). Samples were incubated at 56°C for 1 h and then centrifuged for 5 min at 300 × g at 4°C to pellet insoluble debris. Supernatants were transferred into new microcentrifuge tubes and 400 μl of buffer AL was added. Samples were vortexed briefly and incubated at 56°C for 10 min. Each sample was then mixed with 400 μl of 100% EtOH, vortexed briefly, and applied to the DNeasy silica columns according to manufacturer instructions. After washing columns according to manufacturer's instructions, DNA was eluted in 100 μl of buffer AE. Quality and quantity of DNA was assessed as described above for the adult tissues.

#### mtDNA and gDNA qPCR assays

Primers for qPCR were designed using default settings in the IDT PrimerQuest tool (http://www.idtdna.com/Primerquest/) with the mitochondrial gene *NADH dehydrogenase 4* (*ND4*) or nuclear *insulin-like growth factor-1 receptor* (*IGFR1*, NCBI Reference Sequence: XM_014003519.1) genes as inputs (Table S4). The amplification specificity of each gene/primer set was verified by cloning amplicons generated from *A. limnaeus* embryo tissues into the pGEM-T Easy Vector (Promega, Madison, WI, #A1360), transformation into JM109 High-efficiency cells (Promega, #L2001), and Sanger DNA sequencing the plasmid inserts as previously described (Wagner and Podrabsky, 2015). Purified plasmids with verified PCR inserts for either *ND4* or *IGFR1* were used as standards for qPCR assays. qPCR reactions (20 μl total volume) were set up in triplicate using SsoAdvanced Universal SYBR Green Supermix (Bio-Rad, Hercules, CA, USA, #172-5270) and consisted of 25 ng total DNA, 10 μl of 2X SsoFast Probes Supermix, and 500 nmol l^−1^ of forward and reverse primers. Assays were set up in clear 96-well semi-skirted PCR plates (Hard-Shell High-Profile PCR plates, Bio-Rad, #HSS-9601) with optical flat caps (Bio-Rad, #TCS-0803). All qPCR reactions were carried out in the Stratagene Mx3005P thermocycler (Agilent Technologies, Santa Clara, CA). Standard curves were generated using a serial 1:10 dilution of amplicon-specific plasmid copies (1 ng–1e^−7^ ng for *ND4* and 1e^−1^–1e^−7^ for *IGFR1*). Reactions were initially heated at 95°C for 5 min to activate the DNA polymerase and subsequently thermocycled for 35 cycles by denaturation at 95°C for 30 s and annealing/elongation at 60°C for 30 s. Fluorescence readings (excitation 492 nm, emission 516 nm) were taken at the end of each elongation step. Quantification cycle (C_q_) thresholds were set automatically in Stratagene MxPro software (v.4.10, 2007, Agilent Technologies) using adaptive baseline, moving average, and amplification-based threshold settings. Although rarely necessary, thresholds were manually adjusted to improve standard curve best-fit regressions. Reaction efficiencies of *ND4* or *IGFR1* (E_gene_) were estimated using the standard curve slopes such that E_gene_ = 10^(−1/*slope*)^ (Pfaffl, 2001). Assays were validated by visualizing qPCR end products via gel electrophoresis in addition to observation of a single melting dissociation curve.

### Citrate synthase activity assays

Embryos were treated, sampled, weighed, and flash-frozen for CS activity assays as described for the anoxia qPCR assays, except replicates consisted of 7 pooled individuals for all stages but DII, which consisted of 9 individuals per replicate. Embryos previously frozen in 1.5 ml microcentrifuge tubes were diluted 1:10 (w:v) in ice-cold 10 mmol l^−1^ HEPES (pH 7.5 at 25°C) and homogenized on ice using a Teflon pestle. Crude homogenates were centrifuged at 300 × *g* for 5 min at 4°C to pellet insoluble debris. Supernatants were transferred to new tubes and stored at −80°C until assays were performed. Total citrate synthase enzymatic activity was determined spectrophotometrically at 25°C (PharmaSpec 1700, Shimadzu, Kyoto, Japan) by measurement of absorbance at 412 nm as previously described (Torres and Somero, 1988; Chennault and Podrabsky, 2010). Assays were performed with 1 to 2 technical replicates for each sample. Total CS activity was measured in 1 ml 50 mmol l^−1^ imidazole (pH 7.7 at 25°C) buffer containing 15 mmol l^−1^ MgCl_2_, 0.1 mmol l^−1^ DTNB, and 0.1 mmol l^−1^ acetyl coenzyme A. Background activity for each sample was determined by addition of 10 μl embryo homogenate and measurement of change in absorbance for 2 min. Assays were then initiated by addition of 25 μl of 0.7 mmol l^−1^ oxaloacetic acid (in imidazole buffer as described above) and change in absorbance was recorded for 4 min. Activity slopes were calculated by linear regression in GraphPad Prism (v5.0, La Jolla, CA, USA) using absorbance values for the last 60 s of the background activity and the first 60 s following addition of oxaloacetic acid. Final CS activity slopes were determined by subtraction of background activity from the total activity following addition of oxaloacetate to yield CS-specific activity. Activities are expressed in international units (IU, μmol oxaloacetate min^−1^ embryo^−1^).

### Statistical analysis

Statistical analysis for qPCR and CS activity data were performed using GraphPad Prism v5.0. Differences in relative copy number or CS activity were calculated using one-way Analysis of Variance (ANOVA) followed by Tukey's multiple comparison test (Tukey's MCT). Statistical significance was determined at *P* < 0.05. Linear regression of normoxic data was also performed using GraphPad Prism v5.0. For qPCR experiments, fold differences in mtDNA (*ND4*) or gDNA (*IGFR1*) copy number in PDII embryos were calculated relative to DII samples using the efficiency corrected ddC_q_ method (Relative fold difference of gene = ${\text{E}}_{\text{gene}}^{\left(\text{CqDII}-\text{CqPDII}\right)}$, where C_*q*_DII is the C_q_ of the DII sample and C_q_PDII is the C_*q*_ of the PDII sample; Pfaffl, 2001). Prior to statistical analysis of qPCR data by one-way ANOVA, relative mtDNA copy number was normalized to relative gDNA copy number [Normalized *ND4* fold difference = (Relative fold difference in *ND4*)/(Relative fold change in *IGFR1*)] and log_2_ transformed.

## Results and discussion

### Sequence analysis of the *A. limnaeus* mitochondrial genome

#### Coding gene composition and gene structures

**Figure 1:**
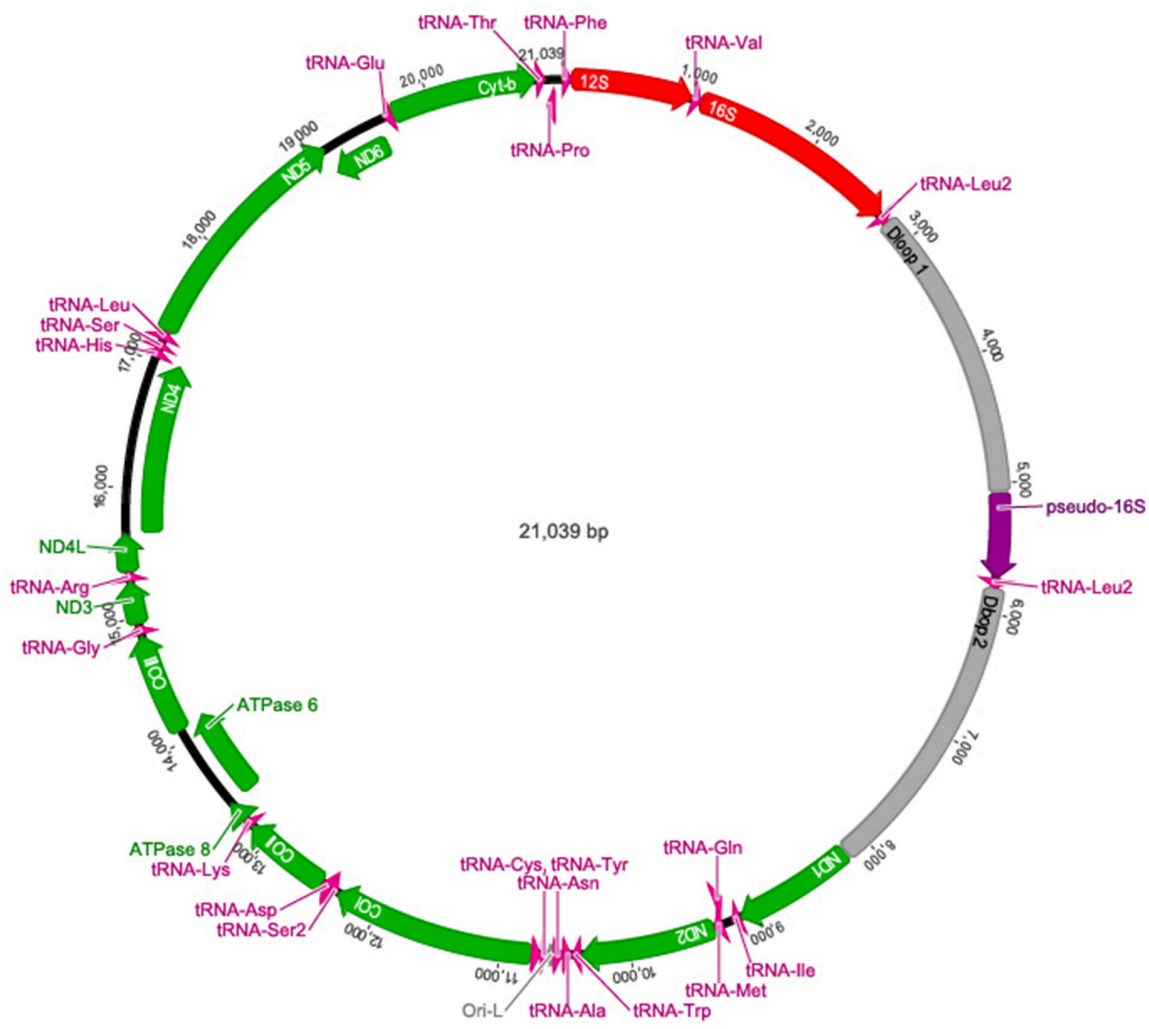
**The complete annotated mtgenome of ***A. limnaeus*****. Protein coding genes are noted in green, tRNAs in pink, D-loops and the origin of light strand replication (abbreviated Ori-L) are in gray, rRNA in red, and the pseudo-16S rRNA in purple.

**Figure 2:**
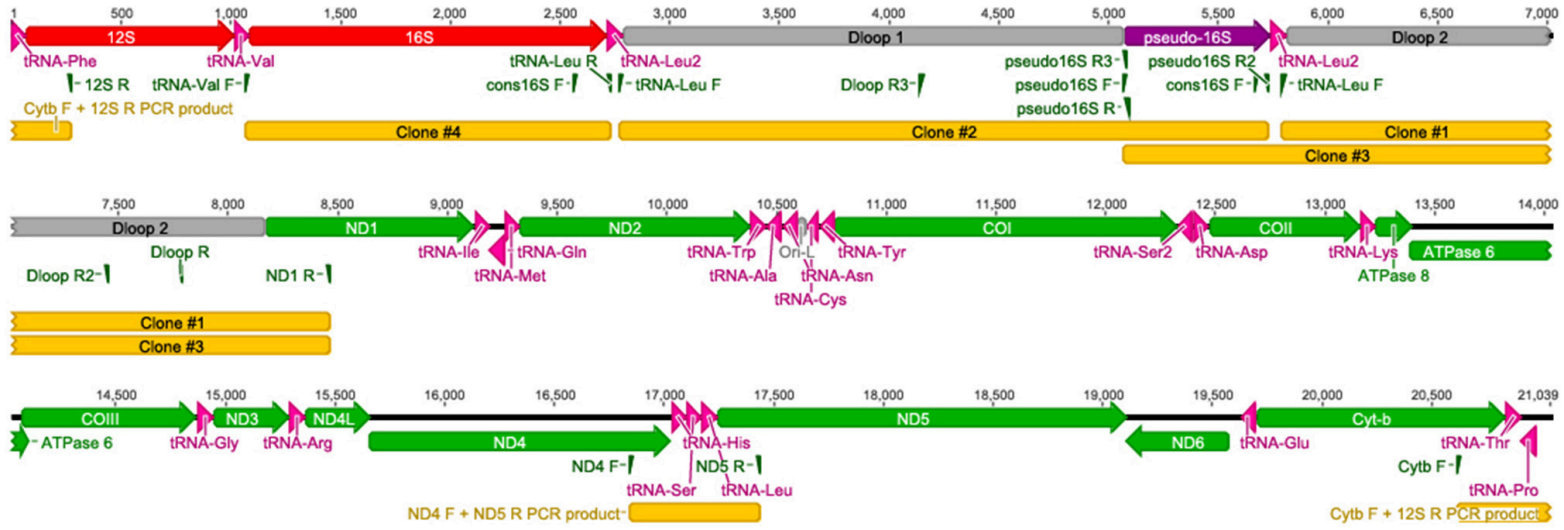
**Primer binding sites for targeted resequencing of the ***A. limnaeus*** mtgenome**. Specific primers were used to resequence parts of the *A. limnaeus* mtgenome. Longer fragments were cloned into vectors for sequencing.

The complete *A. limnaeus* mitochondrial genome contains 21,039 bases, 37.3% GC content, and is circular. MitoAnnotator identified two rRNAs, 21 unique tRNAs, 13 protein-coding genes, and two D-loops (D-loop 1 and D-loop 2) separated by a partial 16S rRNA unit and duplicated tRNA-Leu2 sequence (Figure 1, Table 2). The initial assembly of the *A. limnaeus* mtgenome using only MitoBIM and Illumina reads incorrectly assembled the duplicated D-loop region, as discovered by post-assembly read mapping and identification of breakpoints characteristic of misassembly. Cloned PCR products amplified from around the D-loop region identified the correct sequence order (Figure 2). The correct sequence order was confirmed by mapping of Illumina reads from both forward and reverse libraries with a final average coverage of 9059X (range 1579X–19,520X). A total of 1,705,832 reads mapped with 85% of bases mapping with quality >Q20. The resulting mtgenome assembly has been deposited into NCBI Genbank (KX371089).

**Table 2:** *****A. limnaeus*** mitochondrial feature table**.

**Gene/element**	**Strand**	**Start position**	**Stop position**	**Size (bp)**	**Start codon**	**Stop codon**	**3′^a^**	**TM^b^**
tRNA-Phe	H	1	69	69	–	–	0	–
12S rRNA	H	70	1015	946	–	–	0	–
tRNA-Val	H	1016	1084	69	–	–	0	–
16S rRNA	H	1085	2718	1634	–	–	0	–
tRNA-Leu2	H	2719	2791	73	–	–	0	–
D-loop 1	–	2792	5075	2284	–	–	0	–
pseudo16S	H	5076	5739	664	–	–	0	–
tRNA-Leu2	H	5740	5812	73	–	–	0	–
D-loop 2	–	5813	8175	2363	–	–	0	–
ND1	H	8176	9123	948	GTG	TAA	2	8
tRNA-Ile	H	9126	9193	68	–	–	−1	–
tRNA-Gln	L	9193	9263	71	–	–	−1	–
tRNA-Met	H	9263	9332	70	–	–	0	–
ND2	H	9333	10383	1051	ATT	T–	0	2
tRNA-Trp	H	10384	10457	74	–	–	0	–
tRNA-Ala	L	10458	10526	69	–	–	1	–
tRNA-Asn	L	10528	10600	73	–	–	0	–
O_L_	-	10601	10636	36	–	–	0	–
tRNA-Cys	L	10637	10693	57	–	–	2	–
tRNA-Tyr	L	10696	10763	68	–	–	1	–
COI	H	10765	12327	1563	ACG	TAA	2	12
tRNA-Ser2	L	12330	12399	70	–	–	2	–
tRNA-Asp	H	12402	12472	71	–	–	2	–
COII	H	12475	13165	691	GTG	T–	0	3
tRNA-Lys	H	13166	13231	66	–	–	2	–
ATPase-8	H	13234	13401	168	ACG	TAA	−16	1
ATPase-6	H	13386	14108	688	ATG	AGA	−35	7
COIII	H	14074	14868	795	ATG	TAA	6	7
tRNA-Gly	H	14875	14946	72	–	–	6	–
ND3	H	14953	15295	343	ATA	T–	0	3
tRNA-Arg	H	15296	15364	69	–	–	0	–
ND4L	H	15365	15661	297	GTG	TAA	−7	3
ND4	H	15655	17031	1377	ATG	TAA	5	13
tRNA-His	H	17037	17105	69	–	–	2	–
tRNA-Ser	H	17108	17171	62	–	–	3	–
tRNA-Leu	H	17175	17246	72	–	–	2	–
ND5	H	17249	19111	1863	ATA	TAA	−4	16
ND6	L	19108	19575	468	GTG	TAA	54	4
tRNA-Glu	L	19630	19698	69	–	–	4	–
Cyt-b	H	19703	20838	1136	ATG	TA-	0	8
tRNA-Thr	H	20839	20908	70	–	–	−1	–
tRNA-Pro	L	20908	20980	73	–	–	0	–

a *Number of bases until the start of the next gene or feature*.

b *Total number of transmembrane domains in protein coding genes as identified by TOPOS*.

The protein-coding genes included NADH-ubiquinone oxidoreductase (complex I) subunits 1, 2, 3, 4, 5, and 6 (*ND1, ND2, ND3, ND4, ND5*, and *ND6*), cytochrome *c* oxidase subunits 1, 2, and 3 (*COI, COII, COIII*), cytochrome *b* (*Cytb*), and F_1_F_*o*_-ATP synthase subunits (*ATPase-6* and *ATPase-8*). Identical to the mtgenomes of other species within the Order Cyprinodontiformes used in this study, most identified genes and RNAs were determined to be encoded on the heavy (H, purine rich) strand, aside from the *ND6* gene and eight tRNAs which are encoded on the light (L, pyrimidine rich) strand (Iwasaki et al., 2013). As expected, TOPCONS was able to predict transmembrane domains for all putative coding genes based on translated structure (Table 2).

The overall gene nucleotide composition of the *A. limnaeus* mtgenome is similar to other Cyprinodontiform fishes examined (Table 1). Also similar to the other Cyprinodontiformes, the *A. limnaeus* coding genes that are transcribed on the H strand are depleted of guanine at the third codon position (Figure S1A). This bias against guanine at the third codon positions is not as apparent in *ND6* (Figure S1B), which is transcribed on the L strand. Overall, the *A. limnaeus* coding genes show negative skew for AT [skew: (A−T)/(A+T)] and GC [skew: (G−C)/(G+C)], indicating a bias toward the pyrimidines T and C in all coding genes except for *ND6* (Figure S2). The top five most frequent codons for the aplocheloid fish are listed in Table S5. These five species share three of the top five most frequently used codons in their respective mtgenomes.

Five SNPs were identified at 10% frequency or higher, with four occurring in the 16S rRNA and one predicted to cause a synonymous amino acid change in *ND4L* (Table 3). The four SNPs occurring in the 16S rRNA appear to occur as a single haplotype, as suggested by nearly identical frequency of occurrence of the SNPs at this site, as well as simultaneous presence of all four SNPs in the reads that map to the region. The shared frequency between the 16S rRNA SNPs and the *ND4L* SNP indirectly suggest that there may be an alternative mitochondrial haplotype occurring at ~25% in the read pool that contains the five identified SNPs.

**Table 3:** **Nucleotide polymorphisms detected in the ***A. limnaeus*** mtgenome using the Geneious variant caller**.

**Gene**	**Position(s)**	**Polymorphism Type^a^**	**Variant Frequency**	**Length^b^**	**Change(s)**	**Coverage**
ND4L	15,751	SNP (transversion)	25.0%	1 bp	T 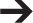 A	9616X
16S	1584; 1587; 1588; 1591	MNV (transitions)	25.1%-25.7%	8 bp	G 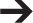 A; G 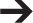 A; T 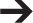 C; T 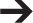 C	7222X–7507X

*Variants were called with a threshold of ≥ 10% occurrence in the Illumina reads*.

a *SNP, single nucleotide polymorphism; MNV, multinucleotide polymorphism*.

b *Total length in bp that the polymorphism spans over the reference sequence*.

#### Cross-species sequence divergence of coding genes

**Figure 3:**
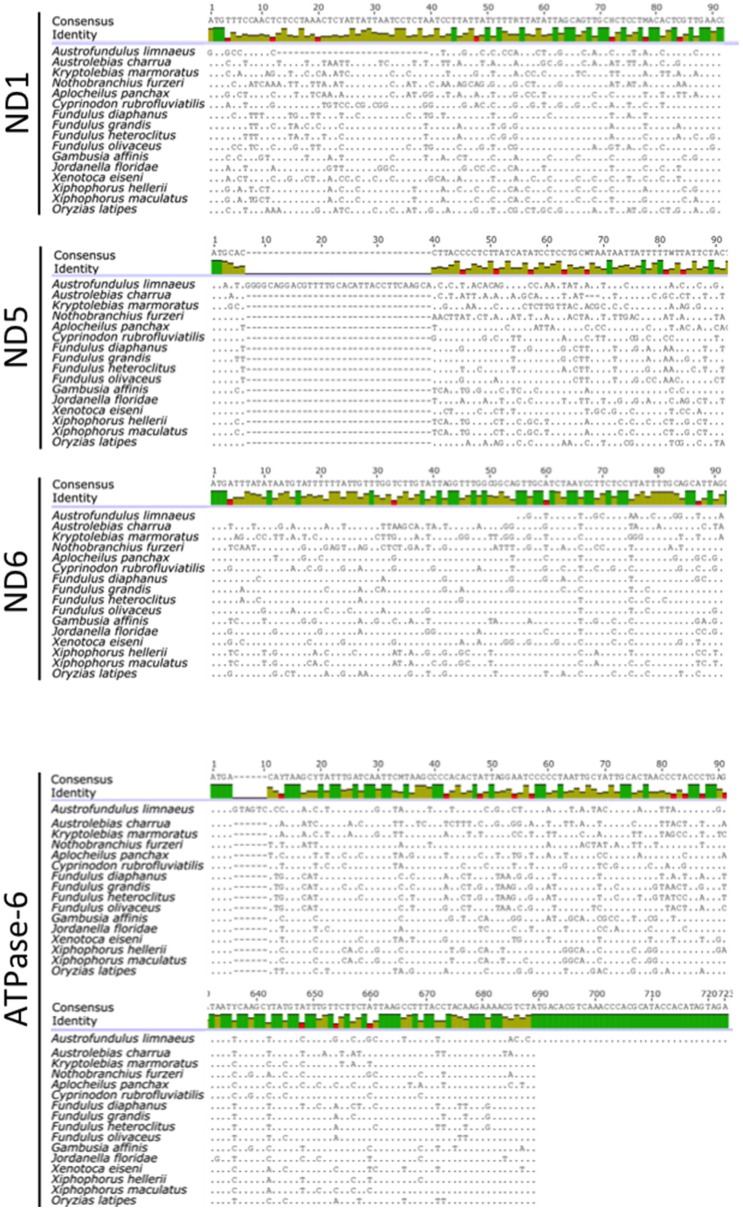
**Alignments of ***A. limnaeus ND1***, ***ND5***, ***ND6***, and ***ATPase-6*** genes to other species**. When compared to other species, there are apparent multinucleotide deletions in the 5′ region of *A. limnaeus ND1* (27 bp) and *ND6* (54 bp). *ND5* appears to have a multinucleotide insertion near the 5′ end when compared to multispecies consensus (33 bps), while *ATPase-6* is extended by several nucleotides at the 5′ end (6 bp) and 3′ end (34 bp).

Although the total length of the *A. limnaeus* mtgenome is larger than other species used in this study, the lengths of coding genes are comparable, with exceptions for *ND1, ND5, ND6*, and *ATPase-6* (Figure 3). The *A. limnaeus ND1* gene is shorter near the 5′ by 27 nucleotides (alignment positions 13–39) and also differs by having a “GTG” start codon rather than “ATG.” *ND5* is longer near the 5′ end by 33 nucleotides (alignment positions 7–39) and has an “ATA” start codon as opposed to the overall consensus of “ATG.” *ND6* is 5′ truncated by 54 nucleotides (alignment positions 1–54) and has a “GTG” start codon instead of “ATG.” Finally, *ATPase-6* differs at both 5′ and 3′ ends, with a small insertion of 6 nucleotides at the 5′ (alignments positions 5–10) and an extension of 34 nucleotides at the 3′ end compared to other species. NCBI BLASTx or BLASTn searches of the extended sequence in *ATPase-6* did not return significant results. These unique gene features do not have clear implications for the mitochondrial physiology of *A. limnaeus*, but the number of differences, and the clustering of the differences in complex I and V suggest the potential for functional significance.

**Figure 4:**
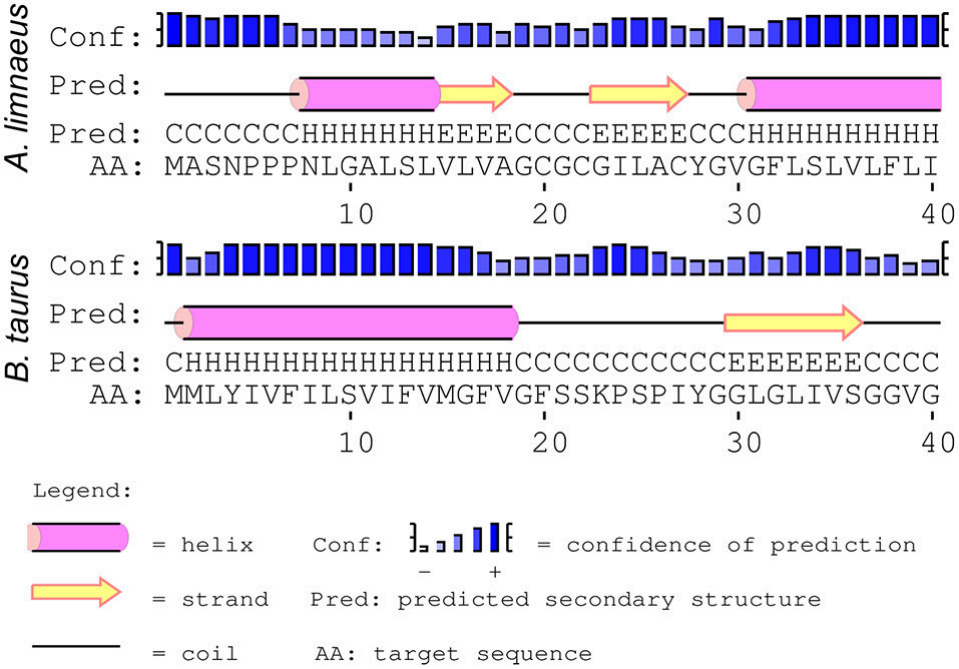
**Comparison of PSIPRED secondary structure results for ***A. limnaeus*** and bovine ND6**. The secondary structure predictions of ND6 in the two species suggests that the *A. limnaeus* protein may be lacking a complete first helix when compared to bovine.

Seven mitochondrial-encoded subunits form the hydrophobic core of complex I, which participates in essential functions such as forming a channel for ubiquinone to move out of the lipid bilayer and into the protein complex and proton transfer (Hirst, 2013). Importantly, the majority of ROS production from ETC activity is thought to occur in complex I (Stowe and Camara, 2009; Quinlan et al., 2012). PSIPRED secondary structure predictions of *A. limnaeus* ND1 and ND5 did not reveal major structural changes when compared to their bovine homologs. However, the first transmembrane helix (ND6-TMH1) of *A. limnaeus* ND6 appears to be incomplete or absent when compared to bovine ND6 (Figure 4). Bovine ND6-TMH1 is believed to be closely associated with other transmembrane helices in ND6 and ND4L. Currently there are no other known instances of a vertebrate missing ND6-TMH1, but the possible functional relevance of these missing residues is unknown (Vinothkumar et al., 2014; Judy Hirst, pers. comm.).

Similar to the structural changes in ND6, the effect of the extended N- and C-termini of the ATPase-6 gene when compared to other fish species is not immediately clear. ATPase-6 comprises the a subunit of complex V (the F_o_ subunit of the F_1_F_o_ATP synthase), which is the complex primarily known for its role as an ion channel that couples proton flux from the inter-membrane space into the matrix with ATP synthesis through interactions with the F_1_ subunit of the ATP synthase complex (Jonckheere et al., 2012). Tight regulation of ATP synthesis, and prevention of ATP consumption during dormancy associated with diapause or anoxia is clearly important (St-Pierre et al., 2000). Making inferences on the possible functional implications of these sequence changes is difficult due to a lack of detailed information on the crystal structure of the F_o_ complex (DeLeon-Rangel et al., 2013). The role of the rather long extension of the C-terminus in ATPase-6 is intriguing and potentially offers the possibility of altered interactions with other subunits of the complex or with proteins in the intermembrane space.

Because complexes I and V both contain several essential and accessory subunits encoded by the nucleus, it will be interesting to see if these other subunits also contain structural changes that are divergent from other fish species. Additionally, confirmation of these complex I and complex V subunit features in other populations of *A. limnaeus* and exploration of the functional consequences of these features may be a fruitful area for future studies.

#### Non-coding and pseudo16s region

**Figure 5:**
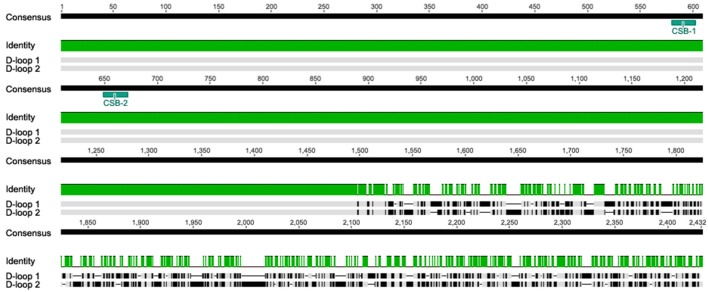
**Pairwise alignment of mitochondrial Alim-D-loop 1 and Alim-D-loop 2 using MUSCLE**. The alignment reveals a 1497 bp segment with 100% similarity in the 5′ region, with a highly divergent region toward the 3′ end. The duplicated regions also contain duplicated CSB-1 and CSB-2.

**Figure 6:**
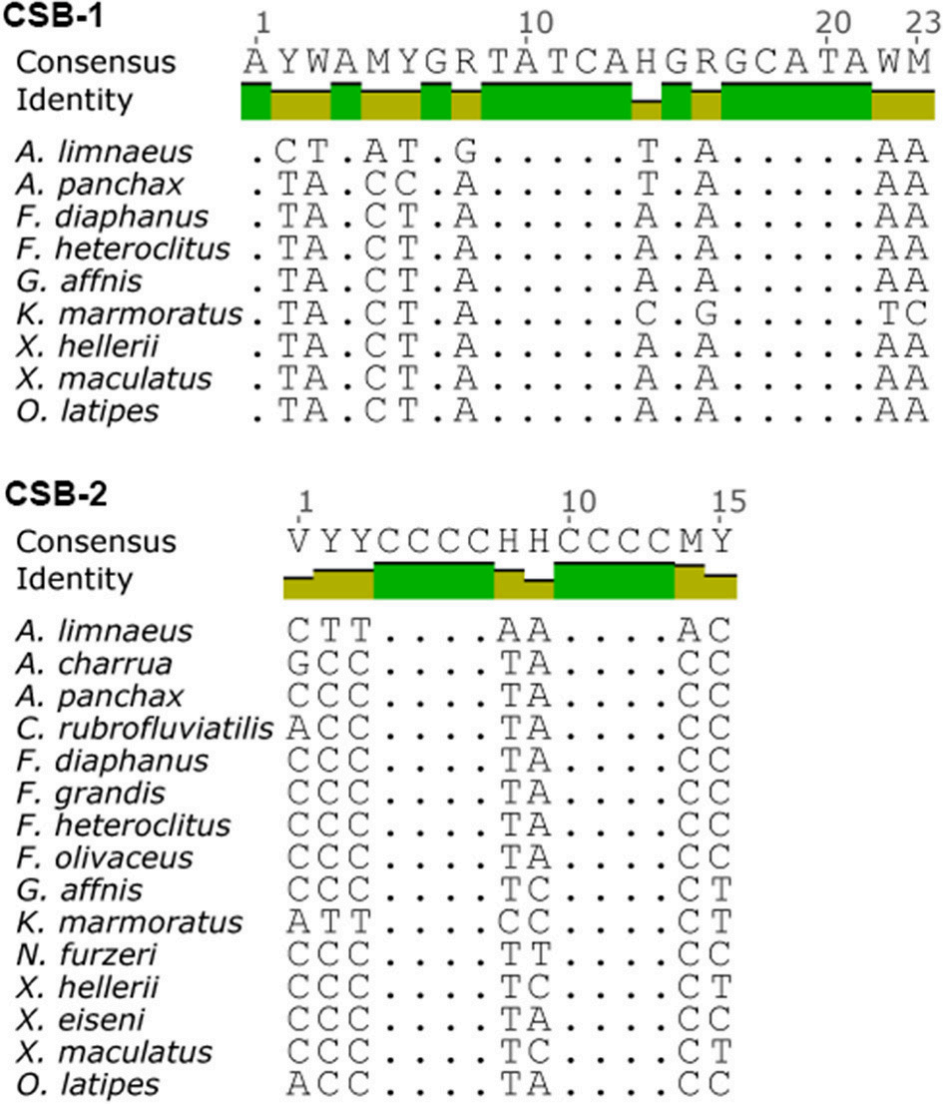
**Consensus motifs of CSB-1 (top) and CSB-2 (bottom) found in the D-loops of species used in this study**. CSBs were found by searching the mtgenomes using conserved motifs based on previous work by Broughton et al. (2001).

Sequence alignment of the two *A. limnaeus* D-loops (Alim-Dloop 1 and Alim-Dloop 2) identified a 1497 bp region with 100% pairwise identity with the remaining 3′ sections appearing to be evolving independently (Figure 5). Both Alim-D-loop 1 and Alim-D-loop 2 contain a high proportion of AT repeats, the most frequent of which is the motif “TAA,” significantly clustered in the unshared portion of D-loop sequences (Figure S3). After alignment of D-loops from several species, two putative CSBs were identified in *A. limnaeus* (CSB-1 and CSB-2), and these CSBs were discovered in both Alim-D-loop 1 and Alim-D-loop 2 based on the conserved motif previously identified by Broughton et al. (2001) (Figure 6). Aside from the three aplocheloid species (*Nothobranchius furzeri, Kryptolebias marmoratus* and *A. limnaeus*) a single D-loop is found between tRNA-Phe and tRNA-Pro, suggesting that this may be the ancestral positioning of the D-loop. In *A. limnaeus*, the non-coding region between tRNA-Phe and tRNA-Pro has been reduced to 59 bp (referred to as pseudoDloop from this point forward), several hundred bps smaller than the other species examined. NCBI blast of this short region did not yield significant results, nor did the sequence have high identity to any other region in the *A. limnaeus* mtgenome. Although sequence and length of the D-loop region can be highly variable between species, possibly due to relaxed selection (Lee et al., 1995), the presence of large D-loop translocations or duplications appears to be uncommon in fishes. Duplicate D-loops have been previously described in snakes (Kumazawa et al., 1996, 1998; Dubey et al., 2012), birds (Abbott et al., 2005; Morris-Pocock et al., 2010), and fish (Lee et al., 2001; Li et al., 2015), but the physiological implications of having repositioned and/or duplicated D-loops are unclear. In snakes, there is evidence that both regions are able to function in replication and transcription processes (Jiang et al., 2007). Similar to the D-loop duplication found in *K. marmoratus* (Lee et al., 2001), the snake duplicated D-loop regions flank both the 12S and 16S rRNAs. Jiang et al. (2007) proposed that having dual regulatory regions might allow for differential regulation of mitochondrial rRNA and mRNA, but this hypothesis has yet to be tested. In typical mammalian mitochondria, different transcriptional binding sites allow for differential transcription of rRNA and mRNAs, with one polycistronic molecule transcribing almost the entire H-strand, and the other stopping after transcribing through the 12S and 16S rRNAs (Fernández-Silva et al., 2003). Fernández-Silva et al. (2003) showed that the incomplete polycistronic RNA containing the 16S and 12S rRNA is transcribed ~20 times more frequently than the polycistronic RNA covering nearly the entire H strand, suggesting that rRNA abundance is an important factor in mitochondrial function. Interestingly, H-strand transcription starting from the repositioned D-loop in *A. limnaeus* would cause the rRNAs to be transcribed near the end of the polycistronic molecule. This implies that either a new transcriptional start site has evolved upstream of the rRNAs, or that all of the H-strand protein coding and tRNA genes would be transcribed along with rRNAs after every round of transcription. Coordinated expression of the entire H-strand may be advantageous to *A. limnaeus* by allowing for rapid and simultaneous expression, or depression, of tRNA, rRNA, and mRNA. The presence of CSB-2, thought to be important in mitochondrial RNA transcription stabilization (Xu and Clayton, 1995; Fernández-Silva et al., 2003; Pham et al., 2006), in both D-loops suggests that H-strand RNA polymerase binding sites may also be duplicated, perhaps allowing for increased transcription rates of the H-strand. Loss of a canonical D-loop between tRNA-Phe and tRNA-Pro has been previously described in the tongue sole, *Cynoglossus semilaevis*, whereby the D-loop occurs in between the *ND1* and tRNA-Gln genes. Thus, it appears fish mitochondrial genomes are able to tolerate diverse translocations of the D-loop while still retaining function.

**Figure 7:**
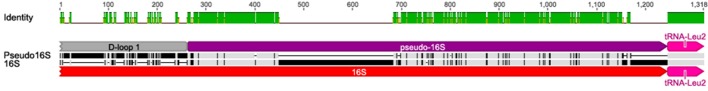
**Pairwise MUSCLE alignment of part of 16S rRNA to the pseudo-16S rRNA region in the ***A. limnaeus*** mtgenome**. The pseudo16S region is located between Alim-D-loop 1 and Alim-D-loop 2. The alignment reveals regions of high similarity between the 16S rRNA and the pseudo-16S. Relative to the reference mtgenome sequence, the pseudo16S region shown here is from positions 4814 to 5812 and the 16S region is from 2227 to 2791.

**Figure 8:**
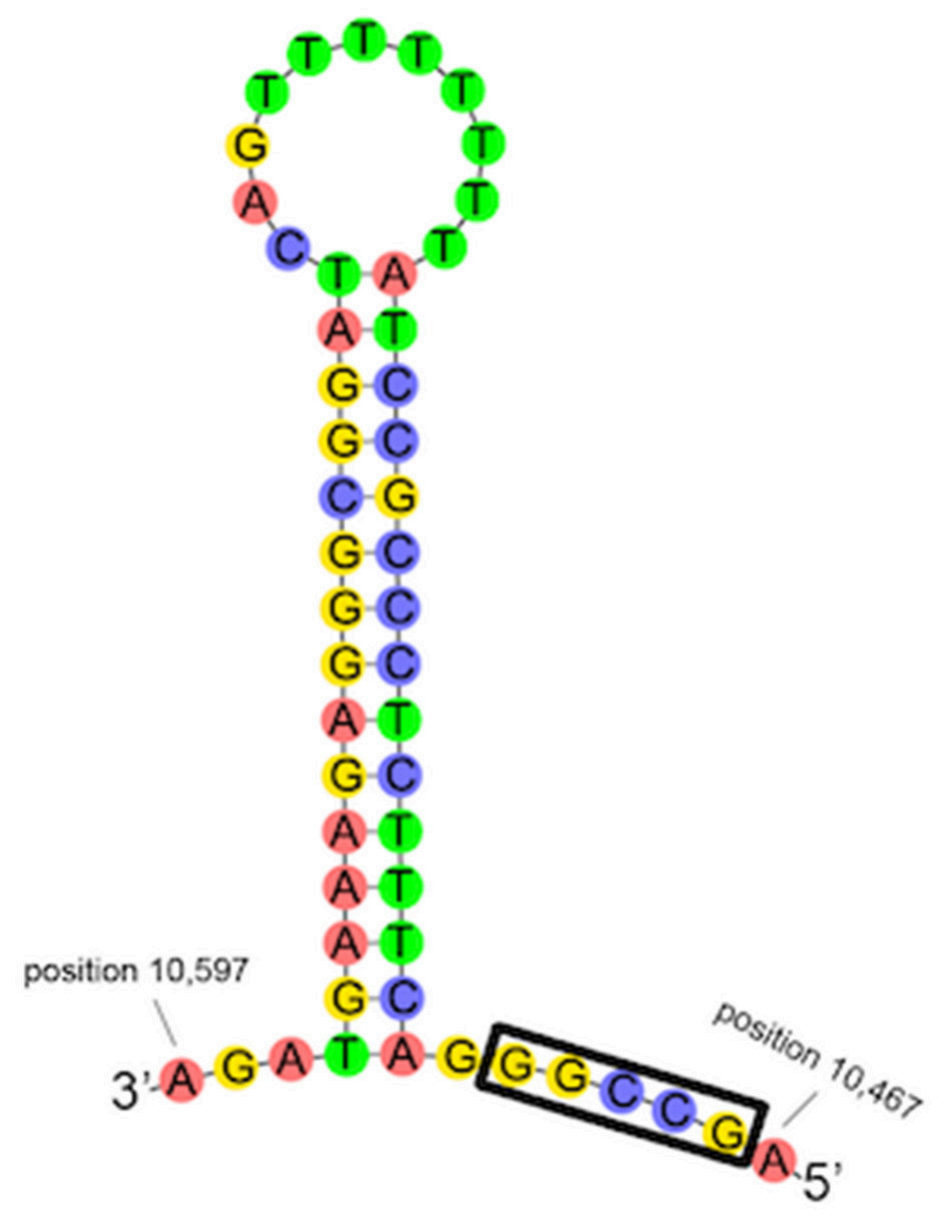
**Predicted folding structure of ***A. limnaeus*** origin of light-strand replication (Ori-L) using Geneious**. The sequence is located between tRNA-Asn and tRNA-Cys and has a high probability of folding into a stable stem-loop structure. The conserved sequence 3′-GGCCG-5′ is indicated with a black rectangle.

Occurring between D-loop 1 and D-loop 2 is a region with partial homology to the 16S rRNA, including a 178 bp (95.2% identity) and 431 bp (88.5% identity) region, collectively referred to as pseudo-16S (Figure 7). The occurrence of the region between *Cytb* and the 12S rRNA and the occurrence of both the 16S and pseudo-16S regions were confirmed in multiple *A. limnaeus* adult individuals (Figure S4). The putative origin of light-strand replication (Ori-L) is a 36 bp region found between tRNA-Asn and tRNA-Cys and is predicted to fold into a stable hairpin structure (Figure 8; Seutin et al., 1994). The Ori-L also contains the conserved motif 3′-GGCCG-5′ near the 5′ end of the hairpin structure, a sequence thought to interact with replication enzymes to direct light-strand replication (Hixson et al., 1986; Hurst et al., 1999).

#### Mitochondrial tRNAs

**Figure 9:**
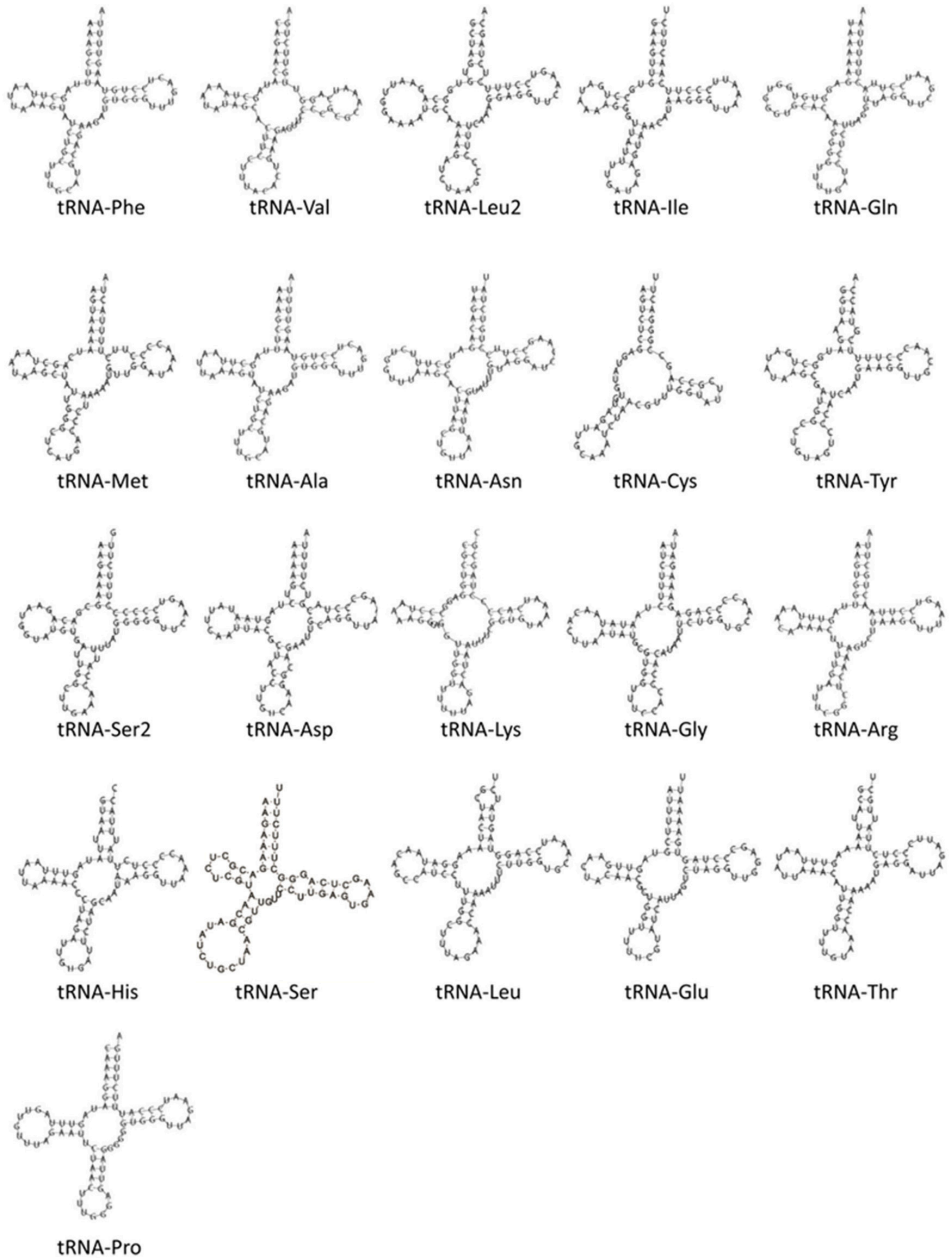
**Predicted secondary structures of identified mitochondrial tRNAs in the ***A. limnaeus*** mtgenome**. The *A. limnaeus* tRNA-Ser was identified by mapping the *N. furzeri* tRNA-Ser to the *A. limnaeus* mtgenome and was folded using Geneious. All other tRNAs were folded by MITOS.

Although nearly all tRNAs were identified and folded into predicted structures (Figure 9), neither MitoAnnotator nor MITOS annotation pipelines were able to predict tRNA-Ser (anticodon AGN) in *A. limnaeus*. Mapping of the *N. furzeri* tRNA-Ser sequence to the assembled *A. limnaeus* mtgenome identified a putitative *A. limnaeus* tRNA-Ser (51.5% pairwise identity). The putative *A. limnaeus* tRNA-Ser has the potential to fold into a cloverleaf structure, as predicted using Geneious. Similar to the previous report for the *A. charrua* mtgenome, the *A. limnaeus* tRNA-Cys is predicted to be the same length as that of *A. charrua* at 57 bp and to lack the D-arm (Figure 9). This feature of tRNA-Cys missing the D-arm [tRNA-Cys(−D)] found in the two South American annual killifishes is not found in any of the species examined here, all of which carry a complete tRNA-Cys [tRNA-Cys(+D)] of typical tRNA length. The *A. limnaeus* mtgenome also contains a duplicated tRNA-Leu2. Although uncommon, duplicated mitochondrial tRNAs have been previously reported, including the Cyprinodontiform *Xenoteca eiseni* (duplicated tRNA-Met; Setiamarga et al., 2008). Initially, the *N. furzeri* mtgenome was thought to contain a duplicated tRNA-Glu but more recently this putative tRNA was suggested to be a pseudogene (Hartmann et al., 2011; Tatarenkov et al., 2015).

### RNAseq mapping predicts mitochondrial transcript polyadenylation sites

**Figure 10:**
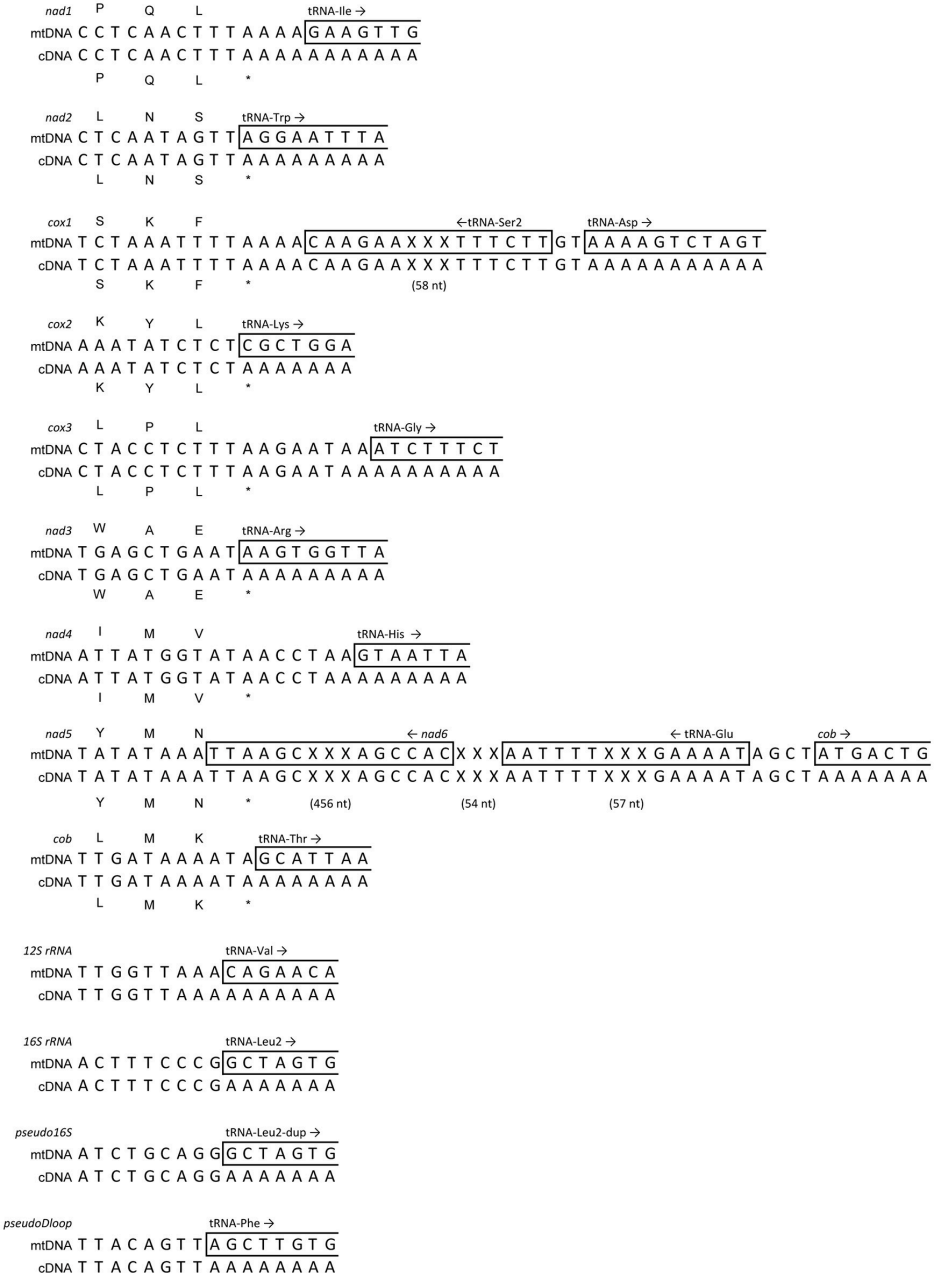
**Predicted polyadenylation sites based on RNAseq mapping onto mitochondrial transcripts**. RNAseq reads were mapped to the *A. limnaeus* mtgenome using Geneious in order to predict polyadenylation sites at the 3′ ends of several coding and non-coding regions. For clarity, some nucleotides in *COXI* and *ND5* have been omitted, replaced with “XXX,” and are noted with the number of nucleotides in parenthesis.

We found a total of 13 regions with polyadenylation signals supported by RNAseq mapping (Figure 10, Supplemental Data Sheet 1). Overall, the mtgenome had an average of 72,697X (s.d. = 100,465.3) with a minimum coverage of 181X and maximum of 909,375X (Figure S5). These data also confirmed the completion of stop codons by post-transcriptional polyadenylation for *ND2, COII, ND3*, and *Cyt-b* genes as predicted by MitoAnnotator (Table 2). In contrast to a similar analysis performed on the Atlantic Cod (*Gadus morhua*) that utilized deep RNA sequencing (Coucheron et al., 2011), we found that *ATPase-8* and *ATPase-6* do not share a single bicistronic transcript but instead the polyadenylation site occurs downstream following *COIII*. Additionally, the Atlantic Cod *ND5* was reported to contain a stop codon immediately at the end of the coding region, while the *A. limnaeus ND5* transcript is not polyadenylated until after the antisense *ND6* and tRNA-Glu are transcribed, 592 bases downstream of the end of *ND5*. This long 3′ trailer following *ND5* is more similar in length to the trailer found in humans than in the Atlantic Cod (Temperley et al., 2010). Similar to previous reports for the Atlantic Cod and humans, we found evidence for both mitochondrial rRNAs being polyadenylated (Dubin et al., 1982; Bakke and Johansen, 2002). Surprisingly, we observed a strong signal for polyadenylation of the presumed ancestral D-loop region (pseudoDloop) between tRNA-Pro and tRNA-Phe. However, nucleotide BLAST searches of this region did not yield homology to any known sequences in the nucleotide database and did not have significant similarity to any other regions in the *A. limnaeus* mtgenome. We also observed a clear polyadenylation signal at the 3′ end of the pseudo16S, suggesting that the 3′ end of the pseudo16S is still processed during mitochondrial transcript maturation.

### Phylogenetics of the cyprinodontiformes using mtDNA

**Figure 11:**
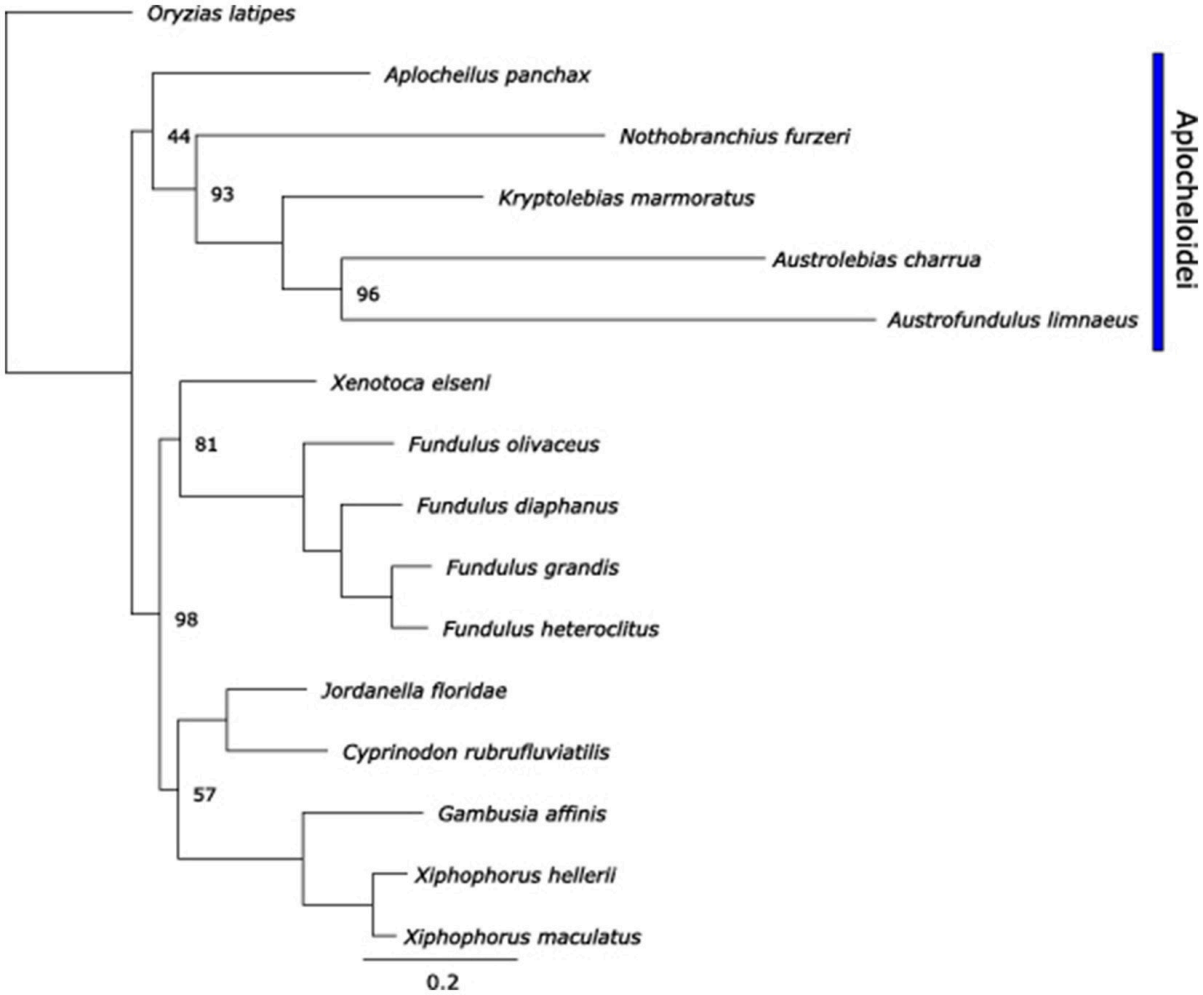
**Maximum-likelihood phylogenetic tree of species used in this paper using RAxML**. Node bootstrap values are 100% unless indicated otherwise. Scale bar is nucleotide substitutions per site.

The use of mtDNA for phylogenetic inference has been a common practice for several decades. Although more recently the validity of such inferences that rely only on a single molecule with very important physiological roles has come into question (Ballard and Whitlock, 2004), phylogenetic inference based on mtDNA is still commonly used for species for which nuclear genome evidence is sparse. Use of all coding and tRNA sequences of mtgenomes has been shown to improve bootstrap support in teleost molecular phylogenies, and thus may provide improved phylogenetic inferences (Miya and Nishida, 2000). ML phylogenetic reconstruction using the concatenated supergenes revealed a similar topology to that found by Tatarenkov et al. (2015). As expected, *Aplocheilus panchax, N. furzeri, K. marmoratus, A. charrua*, and *A. limnaeus* form a single clade representative of the Aplocheiloidei superfamily, with the other Cyprinodontiformes forming the Cyprinodontoidei superfamily clade (Figure 11). Within the Aplocheiloidei superfamily, the topology is in general agreement with the multigene phylogenetic tree proposed by Pohl et al. (2015) with a notable exception that within the Family Rivulidae, *Austrolebias* is sister to *Kryptolebias* in the multigene tree, while the mitochondria-only tree identifies *Austrolebias* as sister to *Austrofundulus*. If the multigene species tree is accepted to also represent the phylogenetic history of each species' mitochondria, this would imply that either (1) the tRNA-Cys(−D) was independently derived in *A. limnaeus* and *A. charrua*, or (2) the last common ancestor for *K. marmoratus, A. limnaeus*, and *A. charrua* was heteroplasmic for tRNA-Cys(+D/−D) and later the tRNA-Cys(−D) genotype became fixed in the two annual killifish species. If the mtDNA-only species tree is accepted, the most parsimonious hypothesis would be that tRNA-Cys(−D) first appeared in the last common ancestor for *A. limnaeus* and *A. charrua*. Additional mitochondrial sequences from species in the Family Rivulidae will prove useful for determining when tRNA-Cys(−D) emerged and how common the feature may be.

### mtDNA copy number dynamics and CS activity during normoxia or anoxia

**Figure 12:**
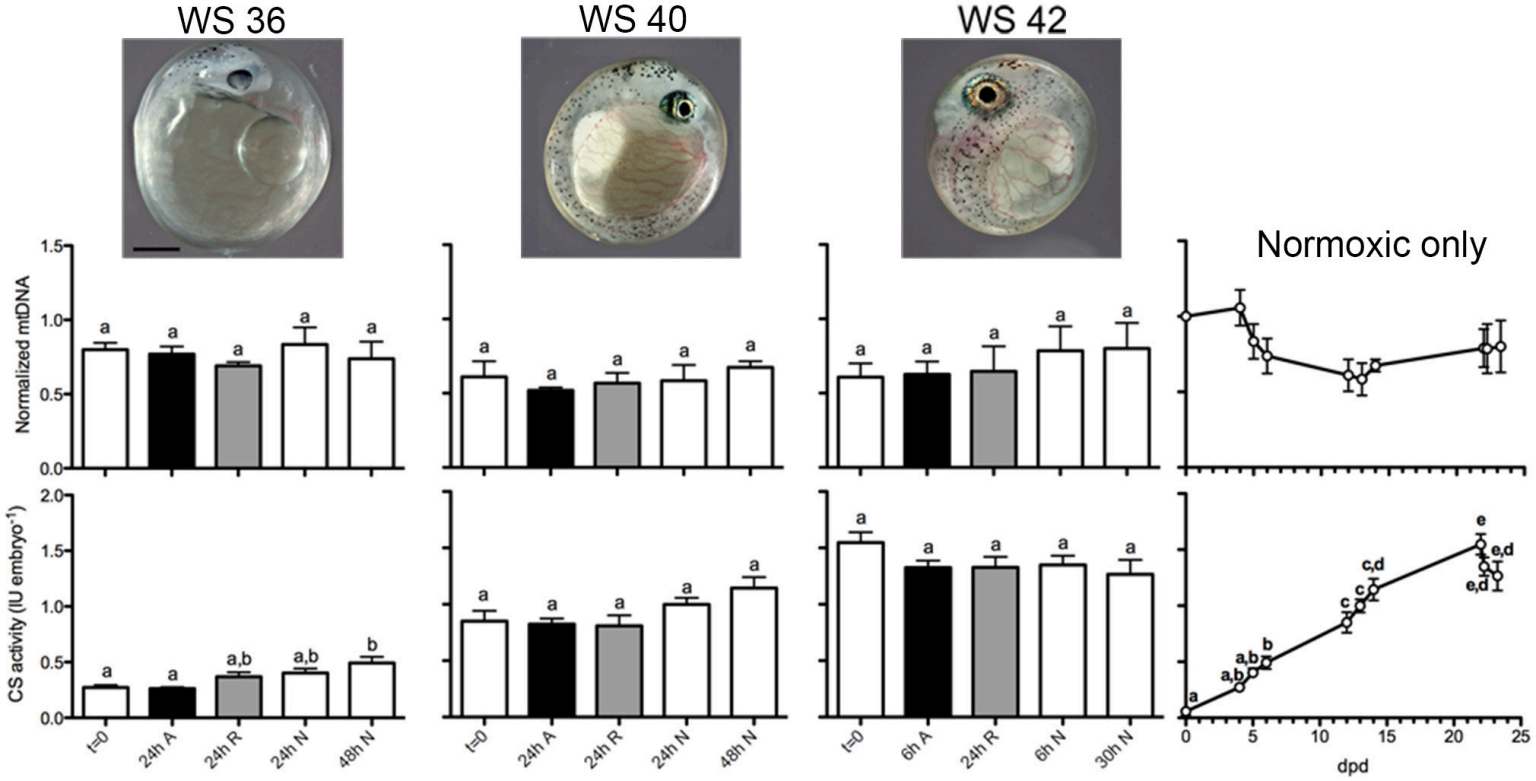
**Relative mtDNA copy number and total CS activity and across development and after anoxia/reoxygenation**. There are no statistically significant differences in relative mtDNA copy number across development or treatment (top graphs). For the CS assays (bottom graphs), different letters above bar graphs correspond to levels of total CS activity that are significantly different within each stage (one-way ANOVA with Tukey's MCT, *P* < 0.05). For the normoxia-only CS line graph data across development, different letters above data points correspond to levels of total CS activity that are significantly different between stages (one-way ANOVA with Tukey's MCT, *P* < 0.05). Embryo photos are from Riggs and Podrabsky, unpublished. Scale bar is 0.5 mm for the embryo photos.

We observed amplification efficiencies in the range of 91.1–94.9% for all qPCR assays. Relative mtDNA copy number did not change significantly in WS 36, 40, or 42 embryos in response to exposure to anoxia or during normoxic recovery (One-way ANOVA, *P* = 0.749 for WS 36, *P* = 0.741 for WS 40, and *P* = 0.75 for WS42; Figure 12). Across normoxic post-diapause II development, the mean relative mtDNA copy number decreased by about 40% in WS 40 embryos when compared to DII, but this trend was not statistically significant (One-way ANOVA, *P* = 0.1288). Because the amplification-based method of qPCR is only able to detect ≥2 fold differences, a more sensitive approach to measuring relative mtDNA may be able to determine if this trend does indeed occur during post-diapause II development.

Exposure to anoxia and up to 24 h of aerobic recovery from anoxia did not result in significant changes in CS activity when compared to *t* = 0 for any of the embryonic stages investigated. These data indicate an overall stabilization of existing CS activity in embryos exposed to anoxia for the three stages tested. In WS 36 embryos, there was an overall statistical significance in CS activity means (one-way ANOVA, *P* = 0.0028). Tukey's MCT identified significant differences between *t* = 0 and 48 h normoxia (*P* < 0.001), and between 24 h anoxia and 48 h normoxia (*P* < 0.001), but no differences were found when 24 h recovery or 24 h normoxia when compared to other treatments within the stage. Thus, in embryos treated at WS 36, the 24 h anoxia treatment is more similar to *t* = 0 than to 48 h normoxia and suggests a slight developmental delay in increased CS activity due to anoxia exposure that is no longer apparent after 24 h recovery. In the WS 40 embryos, there were no statistical differences between means following Tukey's MCT, although there was an overall trend for statistical significance within the stage (one-way ANOVA, *P* = 0.045). In the WS 42 embryos the complex pattern of CS activity associated with entrance into DIII makes the comparison of the anoxia treatments difficult to interpret, and no significant differences were found (one way ANOVA, *P* = 0.31). In contrast to mtDNA copy number across normoxic development, total embryonic CS activity increased linearly starting from DII until 22 dpd at an average rate of 0.0705 IU embryo^−1^ day^−1^ (*R*^2^ = 0.988, Figure 12). This linear trend closely resembles a previous report of total CS activity in developing post-DII *A. limnaeus* embryos by Chennault and Podrabsky (2010). In addition, the increase in total CS activity during post-DII development coincides with a previously reported increase in overall respiratory complex II, IV, and V activity of post-DII *A. limnaeus* embryos (Duerr and Podrabsky, 2010). Following a peak in CS activity at 22 dpd, total CS activity starts to decline in embryos reared in normoxic conditions, presumably as embryos begin to depress aerobic metabolism and enter into DIII (Podrabsky and Hand, 1999). Overall, the changes in CS activity across normoxic post-diapause development were significant (one-way ANOVA, *P* < 0.0001) with individual differences across development following Tukey's MCT shown in Figure 12.

Taken together, these data suggest that there is no direct association between relative mtDNA copy number and aerobic metabolism across normoxic post-DII development in *A. limnaeus*. This trend is similar to zebrafish embryos, whereby the mtDNA/gDNA ratio does not change significantly from 6 h post-fertilization (shield stage) until the end of embryonic development, but the overall rate of aerobic metabolism increases linearly over this same period (Stackley et al., 2011; Artuso et al., 2012). These data imply that as cells replicate during fish embryonic development, on average the number of mtDNA copies per cell remains consistent and does not coincide with the increase in overall aerobic metabolism in a whole embryo. Additionally, these data support the more general hypothesis that total mitochondrial respiration is not regulated at the level of mtDNA copy number (Attardi and Schatz, 1988).

Vertebrate mitochondria are generally highly responsive to changes in oxygen availability (Bickler and Buck, 2007). In mammals, anoxia-sensitive mammalian cells substantially reduce mitochondrial volume and mtDNA copy number when exposed to a hypoxic environment, a result likely achieved through mitophagy (Zhang et al., 2008; Youle and Van Der Bliek, 2012). Reoxygenation after ischemia is typically accompanied by biogenesis of mitochondria, as demonstrated by significant increases in mtDNA content, total cytochrome *c* oxidase Subunit IV (COIV) protein, total CS activity, and total mitochondria number after 24 h of reoxygenation (Yin et al., 2008). It is likely that reactive oxygen species (ROS) mediated signaling associated with reperfusion initiates this response (Lee and Wei, 2005; Li et al., 2012). Whether this biogenesis following reoxgenation is adaptive is uncertain, although it has been suggested to counteract mitochondrial dysfunction by the production of new mitochondria (Rasbach and Schnellmann, 2007; Chen et al., 2011). Nonetheless, these studies suggest that mammalian mitochondria respond dynamically to low oxygen and ischemia/reoxygenation conditions. This response is clearly not shared by embryos of *A. limnaeus* in response to anoxia, as demonstrated by unchanged relative mtDNA content and total CS activity following anoxia or reoxygenation (Figure 12). It should be noted that active development of WS 40 and 42 embryos is absolutely dependent on aerobic respiration (Podrabsky and Hand, 1999). Further, all the post-diapause II stages investigated in this study are comprised of several tissues that are typically oxygen-sensitive, such as a brain and heart, and yet there is no significant change in relative mtDNA content or CS activity in response to anoxia or reoxygenation. Our data suggest that anoxia-tolerant organisms such as *A. limnaeus* do not utilize mitophagy during periods of acute anoxia and do not respond with mitochondrial biogenesis following reoxygenation. Although ROS levels have not yet been measured in *A. limnaeus* embryos following anoxia treatment, it is possible that mitochondrial biogenesis does not occur following reoxygenation because cellular ROS-mediated signaling levels do not become elevated. This is supported by evidence from studies in adult tissues from another anoxia-tolerant vertebrate, the freshwater turtle, where ROS levels are not significantly elevated following anoxia/reoxygenation (Milton et al., 2007). Additionally, there is no evidence for lipid peroxidation during anoxia/reoxygenation in the turtle, further supporting the hypothesis that ROS is effectively controlled in anoxia-tolerant species through unclear mechanisms that may involve antioxidants (Willmore and Storey, 1997; Lutz and Milton, 2004). It would be worthwhile to measure ROS levels in *A. limnaeus* embryos following anoxia/reoxygenation to determine if indeed ROS levels are suppressed, or if there are alternative mechanisms that suppress mitochondrial biogenesis in this species. Additionally, it would be of interest to determine if there is actually mitochondrial turnover (simultaneous biogenesis and degradation) occurring following oxygen insult, whereby the net change in total mitochondria would be near zero, as these data are insufficient to distinguish between mitochondrial turnover and overall stability.

## Author contributions

JW prepared the genomic DNA for sequencing and assembled/annotated the *A. limnaeus* mtgenome with the appropriate software. JW performed the phylogenetic analyses, primer design, PCR for Sanger sequencing, nucleotide alignments, qPCR assays, CS assays, statistical analysis, and wrote the manuscript. FH assisted with validation of qPCR products, preparation and sequencing of clones, performed CS assays, and provided manuscript edits. JEP conceived of the study along with JW and provided manuscript edits and statistical advice.

## Funding

This work was funded by a National Science Foundation (NSF) grant (IOS-1354549) to JEP and research stipend support to FH from the Portland State University Louis Stokes Alliance for Minority Participation (NSF, HRD-140465).

### Conflict of interest statement

The authors declare that the research was conducted in the absence of any commercial or financial relationships that could be construed as a potential conflict of interest.

## Supplementary material

Figure S1 **Overall nucleotide composition of ***A. limnaeus*** mitochondrial coding genes and RNAs**. The nucleotide compositions of the coding genes are separated by codon position. Pos1, codon position 1; Pos2, codon position 2; Pos3, codon position 3; 4fold pos3, codon position 3 that can have any 4 nucleotides in position while still coding for the same amino acid.

Figure S2 **GC and AT skewness across ***A. limnaeus*** mtgenome coding regions**. The *A. limnaeus* mtgenome tends to have bias toward C and T nucleotides in all coding genes except for *ND6*, which has a bias toward G and T. These data do not include stop codons.

Figure S3 **RepFind analysis of Alim-D-loop 1 (top panel) and Alim-D-loop 2 (bottom panel) repetitive motifs**. Both D-loops have a high proportion of repeats high in T and A toward the 3′ end. The RepFind *P*-value indicates the probability of finding the cluster of the specific type of repeats by chance alone.

Figure S4 **Verification of ***A. limnaeus*** mtgenome regions across individuals and tissue types using PCR**. The Cytb F—12S R primer set amplifies the region that contains the canonical D-loop in most other species, thus confirming the loss of the ancestral D-loop in multiple *A. limnaeus* individuals and tissue types. The Cons 16S- F—tRNA-Leu R primer set amplifies regions using primers that are conserved between the 16S rRNA and pseudo-16S, demonstrating the truncated length associated with the pseudo 16S. Abbreviations: Liv, liver; Ov, ovary; WM, white muscle.

Figure S5 **RNAseq coverage of the ***A. limnaeus*** mtgenome**. The coverage ranges from a minimum of 181X to a maximum of 909,375X and covers the reference sequence completely. Reads were mapped using Geneious as described in the main text.

Table S1 **Primer names and sequences used to verify regions of the ***A. limnaeus*** mtgenome**. These primers were used to generate PCR fragments for cloning into the pGEM-T vector, for sequencing the vectors, or both.

Table S2 **Primers used to generate and sequence clones for assembly of the ***A. limnaeus*** mtgenome**. PCR fragments were generated using the listed primer pairs for each clone and were inserted into the pGEM-T vector. Clones were sequenced using the listed primers.

Table S3 **Illumina reads used for assembly and mapping of the ***A. limnaeus*** mitochondrial genome**. Reads were trimmed using Trimmomatic prior to sequence assembly using MitoBIM. SD, standard deviation.

Table S4 **Primers specific to mitochondrial ***NADH dehydrogenase subunit 4*** (***ND4***) or nuclear ***insulin-like growth factor-1 receptor*** (***IGFR1***) used for measurement of relative mtDNA content**. Primer sequences were generated using the IDT PrimerQuest tool.

Table S5 **Top five most used codons in the ***A. limnaeus, A. charrua, N. furzeri, K. marmoratus***, and ***A. panchax*** mtgenomes**. Codon usage was determined using CAIcal. Top codons that are shared by all five species are noted in bold.

Supplemental Data Sheet 1 **RNAseq mapping support for polyadenylation sites in Excel spreadsheet format**. A1, A2, and A3 refer to the first, second, and third sites that mapped as non-reference adenosine.

## Abbreviations

ANOVA: analysis of variance
ATP: adenosine triphosphate
CS: citrate synthase
CSB: conserved sequence-block
DI: diapause I
DII: diapause II
DIII: diapause III
dpd: days post-diapause II
EtOH: ethanol
ML: maximum likelihood
mtDNA: mitochondrial DNA
mtgenome: mitochondrial genome
OXPHOS: oxidative phosphorylation
PDII: post-diapause II
qPCR: quantitative PCR
ROS: reactive oxygen species
SNP: single-nucleotide polymorphism
WS: Wourms' stage.
