# Supplementary figures and images for: Mitochondrial DNA Sequence and Lack of Response to Anoxia in the Annual Killifish Austrofundulus limnaeus
Source: Front Physiol. 2016 Aug 31;7:379. doi: 10.3389/fphys.2016.00379 (PMC5005410; doi:10.3389/fphys.2016.00379)

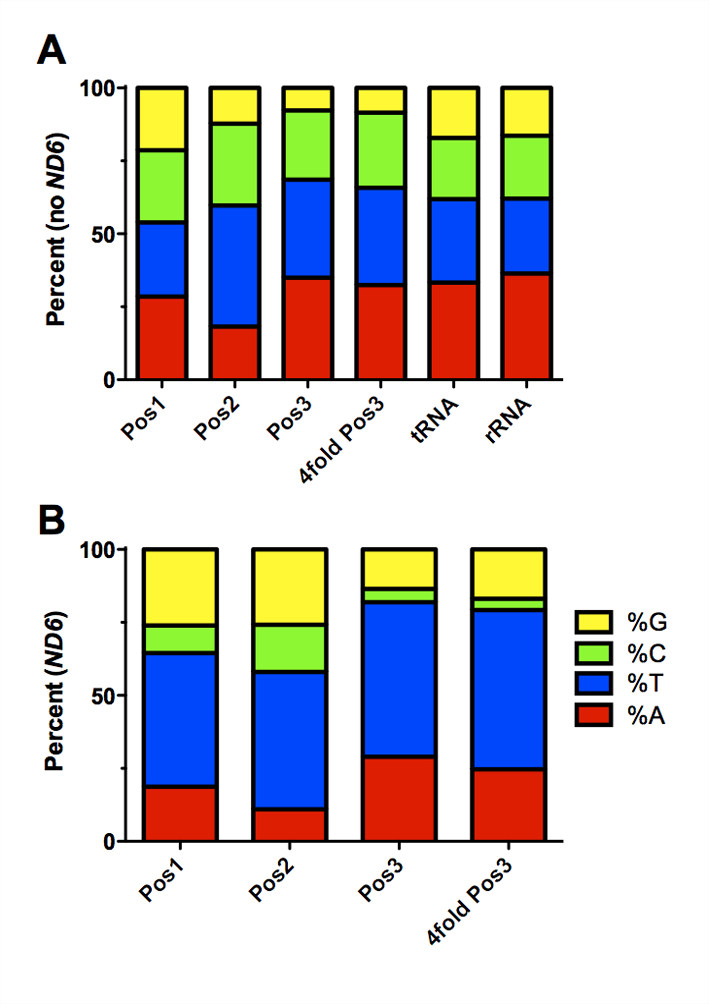

Supplement: Figure S1 — Overall nucleotide composition of A. limnaeus mitochondrial coding genes and RNAs. The nucleotide compositions of the coding genes are separated by codon position. Pos1, codon position 1; Pos2, codon position 2; Pos3, codon position 3; 4fold pos3, codon position 3 that can have any 4 nucleotides in position while still coding for the same amino acid. [file Image1.TIFF]

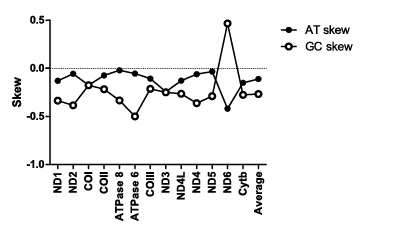

Supplement: Figure S2 — GC and AT skewness across A. limnaeus mtgenome coding regions. The A. limnaeus mtgenome tends to have bias toward C and T nucleotides in all coding genes except for ND6, which has a bias toward G and T. These data do not include stop codons. [file Image2.JPEG]

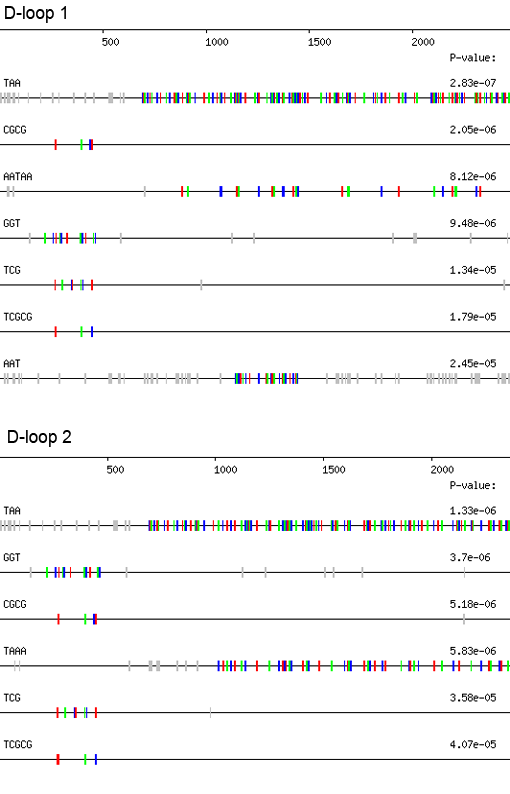

Supplement: Figure S3 — RepFind analysis of Alim-D-loop 1 (top panel) and Alim-D-loop 2 (bottom panel) repetitive motifs. Both D-loops have a high proportion of repeats high in T and A toward the 3′ end. The RepFind P-value indicates the probability of finding the cluster of the specific type of repeats by chance alone. [file Image3.TIFF]

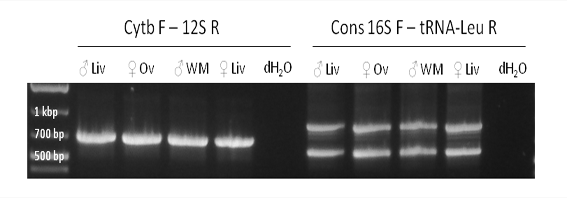

Supplement: Figure S4 — Verification of A. limnaeus mtgenome regions across individuals and tissue types using PCR. The Cytb F—12S R primer set amplifies the region that contains the canonical D-loop in most other species, thus confirming the loss of the ancestral D-loop in multiple A. limnaeus individuals and tissue types. The Cons 16S- F—tRNA-Leu R primer set amplifies regions using primers that are conserved between the 16S rRNA and pseudo-16S, demonstrating the truncated length associated with the pseudo 16S. Abbreviations: Liv, liver; Ov, ovary; WM, white muscle. [file Image4.TIFF]

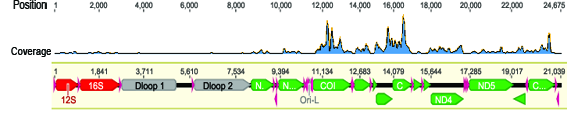

Supplement: Figure S5 — RNAseq coverage of the A. limnaeus mtgenome. The coverage ranges from a minimum of 181X to a maximum of 909,375X and covers the reference sequence completely. Reads were mapped using Geneious as described in the main text. [file Image5.tif]
