# Supplementary material for: Mitochondrial DNA Sequence and Lack of Response to Anoxia in the Annual Killifish Austrofundulus limnaeus
Source: Front Physiol. 2016 Aug 31;7:379. doi: 10.3389/fphys.2016.00379 (PMC5005410; doi:10.3389/fphys.2016.00379)
Supplement: Table S1 — Primer names and sequences used to verify regions of the A. limnaeus mtgenome. These primers were used to generate PCR fragments for cloning into the pGEM-T vector, for sequencing the vectors, or both. [file Table1.DOCX]

| **Primer name** | **Sequence (5’ to 3’)** |
| --- | --- |
| 12S R | GGTCGATCTTACGTTCATTGCT |
| cons16S F1 | GGAGCAATCCAGGTCAGTTT |
| Cytb F | CCCTTCCTCCACACCTCTAA |
| Dloop R | GTTATGTTGGGACTTGGCTTTG |
| Dloop R2 | TTTCACAGGAGTAAGGGCTTT |
| Dloop R3 | ACACTATAAATCTACCAACACCTCT |
| ND1 R | CCTAGGTCTAAATCTGCTAAGGG |
| pseudo16S F | TGTGGTACAATTACATACTTC |
| pseudo16S R | ACATTACGGGGAAGTATGTAATT |
| pseudo16S R2 | CAGATGCTCTTCTTTGGTTCTTTC |
| pseudo16S R3 | GAAGTATGTAATTGTACCACA |
| pUC/M13 F | CGCCAGGGTTTTCCCAGTCACGAC |
| pUC/M13 R | TCACACAGGAAACAGCTATGAC |
| tRNA-Leu F | GGTTCAAGTCCTTTCTCTAGCA |
| tRNA-Leu F2 | GCTAGTGTGGCAGAATTGGA |
| tRNA-Leu R | TCCAATTCTGCCACACTAGC |
| tRNA-Val F | GCGCAAATCAGGTTGTTCTG |
