# Supplementary material for: Mitochondrial DNA Sequence and Lack of Response to Anoxia in the Annual Killifish Austrofundulus limnaeus
Source: Front Physiol. 2016 Aug 31;7:379. doi: 10.3389/fphys.2016.00379 (PMC5005410; doi:10.3389/fphys.2016.00379)
Supplement: Table S2 — Primers used to generate and sequence clones for assembly of the A. limnaeus mtgenome. PCR fragments were generated using the listed primer pairs for each clone and were inserted into the pGEM-T vector. Clones were sequenced using the listed primers. [file Table2.DOCX]

| **Clone #** | **F primer** | **R primer** | **Sequencing primers used** | **Size estimation (bp)^a^** | **Total sequenced^b^ (bp)** |
| --- | --- | --- | --- | --- | --- |
| 1 | tRNA-Leu F | ND1 R | pUC/M13 F, pUC/M13 R, Dloop R, Dloop R2 | 2666-2882 | 2678 |
| 2 | tRNA-Leu F | pseudo16S R2 | pUC/M13 F, pUC/M13 R, Dloop R3, pseudo16S R3 | 2850-3065 | 2966 |
| 3 | pseudo16S F | ND1 R | pUC/M13 F, pUC/M13 R, Dloop R, tRNA-Leu F2 | 3204-3464 | 3401 |
| 4 | tRNA-Val F | tRNA-Leu R | pUC/M13 F, pUC/M13 R | 1660-1805 | 1675 |
| - | ND4 F | ND5 R | ND4 F, ND5 R | 549-637 | 603 |
| - | Cytb F | 12S R | Cytb F, 12S R | 630-738 | 701 |

^a^Size estimation of PCR product when resolved on an agarose gel. Size ranges were estimated by selecting the upper and lower boundaries of the resolved product in GelAnalyser software. For clones, this estimation was done prior to cloning.

^b^Total size of a sequenced clone or PCR product after assembly into a contig in Geneious software.
