# Supplementary material for: Mitochondrial DNA Sequence and Lack of Response to Anoxia in the Annual Killifish Austrofundulus limnaeus
Source: Front Physiol. 2016 Aug 31;7:379. doi: 10.3389/fphys.2016.00379 (PMC5005410; doi:10.3389/fphys.2016.00379)
Supplement: Table S3 — Illumina reads used for assembly and mapping of the A. limnaeus mitochondrial genome. Reads were trimmed using Trimmomatic prior to sequence assembly using MitoBIM. SD, standard deviation. [file Table3.DOCX]

| **Library** | **total bases** | **Average post-trim read length** | **Read length range** | **total reads** | **insert size** | **insert size S.D.** |
| --- | --- | --- | --- | --- | --- | --- |
| Forward pair | 9.42 GB | 94.25 bp | 16-101 bp | 100,000,000 | 170 bp | 20 bp |
| Reverse pair | 9.43 GB | 94.25 bp | 16-101 bp | 100,000,000 | 170 bp | 20 bp |
