# Supplementary material for: Mitochondrial DNA Sequence and Lack of Response to Anoxia in the Annual Killifish Austrofundulus limnaeus
Source: Front Physiol. 2016 Aug 31;7:379. doi: 10.3389/fphys.2016.00379 (PMC5005410; doi:10.3389/fphys.2016.00379)
Supplement: Table S4 — Primers specific to mitochondrial NADH dehydrogenase subunit 4 (ND4) or nuclear insulin-like growth factor-1 receptor (IGFR1) used for measurement of relative mtDNA content. Primer sequences were generated using the IDT PrimerQuest tool. [file Table4.DOCX]

| **Gene** | **Primer sequence (5’ to 3’)** |
| --- | --- |
| ND4 F | CTTATTACTGGGCTCGGAACAT |
| ND4 R | GAGGAGCGTTGGGTAAATAGAG |
| IGFR1 F | CCTGTACAACCTGAGGAACATC |
| IGFR1 R | CTCGGCATCCATAATCAGAGAC |
