# Supplementary material for: Mitochondrial DNA Sequence and Lack of Response to Anoxia in the Annual Killifish Austrofundulus limnaeus
Source: Front Physiol. 2016 Aug 31;7:379. doi: 10.3389/fphys.2016.00379 (PMC5005410; doi:10.3389/fphys.2016.00379)
Supplement: Table S5 — Top five most used codons in the A. limnaeus, A. charrua, N. furzeri, K. marmoratus, and A. panchax mtgenomes. Codon usage was determined using CAIcal. Top codons that are shared by all five species are noted in bold. [file Table5.DOCX]

| ***A. limnaeus*** |  | | | | |
| --- | --- | --- | --- | --- | --- |
| Codon | **ATT** | **TTA** | **TTT** | CTA | ATA |
| Amino Acid | **Ile** | **Leu** | **Phe** | Leu | Met |
| Total codon occurrence | **201** | **190** | **183** | 162 | 163 |
|  | | | | | |
| ***A. charrua*** |  | | | | |
| Codon | **TTT** | **TTA** | **ATT** | CTT | ATA |
| Amino Acid | **Phe** | **Leu** | **Ile** | Leu | Met |
| Total codon occurrence | **266** | **247** | **210** | 182 | 138 |
|  | | | | | |
| ***N. furzeri*** |  | | | | |
| Codon | **TTA** | **ATT** | **TTT** | ATA | CTA |
| Amino Acid | **Leu** | **Ile** | **Phe** | Met | Leu |
| Total codon occurrence | **208** | **185** | **163** | 158 | 157 |
|  | | | | | |
| ***K. marmoratus*** |  | | | | |
| Codon | **ATT** | **TTT** | CTT | **TTA** | CTA |
| Amino Acid | **Ile** | **Phe** | Leu | **Leu** | Leu |
| Total codon occurrence | **193** | **173** | 163 | **152** | 143 |
|  | | | | | |
| ***A. panchax*** |  | | | | |
| Codon | **ATT** | CTA | CTT | **TTA** | **TTT** |
| Amino Acid | **Ile** | Leu | Leu | **Leu** | **Phe** |
| Total codon occurrence | **187** | 178 | 156 | **150** | **138** |
